# Supplementary material for: GaPP2, a multicentre randomised controlled trial of the efficacy of gabapentin for the management of chronic pelvic pain in women: study protocol
Source: BMJ Open. 2018 Jan 31;8(1):e014924. doi: 10.1136/bmjopen-2016-014924 (PMC5879736; doi:10.1136/bmjopen-2016-014924)
Supplement: Author's manuscript [file bmjopen-2016-014924.draft_revisions.pdf]

BMJ Open

BMJ Open is committed to open peer review. As part of this commitment we make the peer review history of every article we publish publicly available.

When an article is published we post the peer reviewers' comments and the authors' responses online. We also post the versions of the paper that were used during peer review. These are the versions that the peer review comments apply to.

The versions of the paper that follow are the versions that were submitted during the peer review process. They are not the versions of record or the final published versions. They should not be cited or distributed as the published version of this manuscript.

BMJ Open is an open access journal and the full, final, typeset and author-corrected version of record of the manuscript is available on our site with no access controls, subscription charges or pay-per-view fees (http://bmjopen.bmj.com).

If you have any questions on BMJ Open's open peer review process please email editorial.bmjopen@bmj.com

BMJ Open

GaPP2: A multi-centre randomised controlled trial of the efficacy of gabapentin for the management of chronic pelvic pain in women: study protocol

Journal:	*BMJ Open*
Manuscript ID	bmjopen-2016-014924
Article Type:	Protocol
Date Submitted by the Author:	26-Oct-2016
Complete List of Authors:	Vincent, Katy; University of Oxford, Nuffield Department of Obstetrics and Gyanecology<br>Baranowski, Andrew ; The National Hospital for Neurology and Neurosurgery , Pain Management Centre<br>Bhattacharya, Siladitya; Aberdeen University, Obstetrics and Gynaecology<br>Birch, Judy; Pelvic Pain Support Network<br>Cheong, Ying; University of Southampton Faculty of Medicine<br>Cregg, Roman; The National Hospital for Neurology and Neurosurgery, Pain Management Centre<br>Daniels, Jane; University of Birmingham, Birmingham Clinical Trials Unit<br>Hewitt, Catherine; University of Birmingham, Clinical Trials Unit<br>Macfarlane, Gary; University of Aberdeen, Epidemiology Group<br>Middleton, Lee; University of Birmingham, Clinical Trials Unit<br>Szubert, Wojciech; University of Edinburgh, MRC Centre for Reproductive Health<br>Tracey, Irene; University of Oxford, Nuffield Department of Clinical Neurosciences<br>Williams, Amanda; University College London, Research Department of Clinical, Educational and Health Psychology<br>Horne, Andrew
**Primary Subject Heading**:	Obstetrics and gynaecology
Secondary Subject Heading:	Evidence based practice
Keywords:	PAIN MANAGEMENT, gabapentin, chronic pelvic pain

SCHOLARONE™ Manuscripts

TITLE PAGE

Title: GaPP2: A multi-centre randomised controlled trial of the efficacy of gabapentin for the management of chronic pelvic pain in women: study protocol

Authors: Katy Vincent[1], Andrew Baranowski[2], Siladitya Bhattacharya[3], Judy Birch[4], Ying Cheong[5], Roman Cregg[2], Jane Daniels[6], Catherine A. Hewitt[6], Gary J Macfarlane[7], Lee Middleton[6], Wojciech Szubert[8], Irene Tracey[9], Amanda C de C Williams[10], Andrew W Horne[8]

[1] Nuffield Department of Obstetrics and Gynaecology, University of Oxford, OX3 9DU, UK.

[2] Pain Management Centre, The National Hospital for Neurology and Neurosurgery, London W1T 4AJ, UK.

[3] Institute of Applied Health Sciences, School of Medicine, Medical Sciences and Nutrition, University of Aberdeen, UK

[4] Pelvic Pain Support Network, Poole, BH17 9AT, UK.

[5] Faculty of Medicine, University of Southampton, SO16 5YA, UK.

[6] Birmingham Clinical Trials Unit, University of Birmingham. B15 2TT. UK

[7] Epidemiology Group, School of Medicine, Medical Sciences and Nutrition, University of Aberdeen, UK

[8] MRC Centre for Reproductive Health, Queen's Medical Research Institute, 47 Little France Crescent, Edinburgh EH16 4TJ

[9] Nuffield Department of Clinical Neurosciences, University of Oxford, OX3 9DU, UK.

[10] Research Dept of Clinical, Educational & Health Psychology, University College London, Gower St, London WC1E 6BT

For peer review only - http://bmjopen.bmj.com/site/about/guidelines.xhtml

Corresponding author:

Dr Katy Vincent

NDOG, University of Oxford

Level 3, The Women's Centre

John Radcliffe Hospital

Oxford

OX3 9DU

Tel: 00 44 1844 221021

Fax: 00 44 1865 769141

email: katy.vincent@obs-gyn.ox.ac.uk

Key Words: Chronic Pelvic Pain; Gabapentin;

Word Count: 3373

For peer review only

For peer review only - http://bmjopen.bmj.com/site/about/guidelines.xhtml

ABSTRACT

Introduction:

Chronic pelvic pain (CPP) affects more than 1 million UK women with associated healthcare costs of £158 million annually. Current evidence supporting interventions when no underlying pathology is identified is very limited and treatment is frequently inadequate. Gabapentin (a GABA analogue) is efficacious and often well tolerated in other chronic pain conditions. We have completed a successful pilot randomised controlled trial (GaPP1) and here describe the protocol for the definitive multicentre trial to assess the efficacy of gabapentin in the management of CPP in women (GaPP2).

Methods and analysis:

We plan to perform a double blind placebo controlled randomised multi-centre clinical trial, recruiting 300 women with CPP from more than 8 NHS hospitals within the UK. After randomisation, women will titrate their medication (gabapentin or placebo) over a 4-week period to a maximum of 2700mg or placebo equivalent and will then maintain a stable dose for a 12 week period. Response to treatment will be monitored with validated questionnaires and co-primary outcome measures of average and worst pain scores will be employed. The primary objective is to test the hypothesis that treatment with gabapentin has the potential to provide a safe, effective and convenient oral treatment and whether it can alleviate pain in women with CPP in the absence of any obvious pelvic pathology.

Ethics and dissemination:

Ethical approval has been obtained from the Coventry and Warwick Research Ethics Committee (REC 15/WM/0036). Data will be presented at international conferences

For peer review only - http://bmjopen.bmj.com/site/about/guidelines.xhtml

and published in peer-reviewed journals. We will make the information obtained from the study available to the public through national bodies and charities.

Trial registration number: ISRCTN77451762

STRENGTHS AND LIMITATIONS OF THIS STUDY

1. This study addresses a key gap in the current evidence regarding the management of chronic pelvic pain when no underlying pathology is identified.

2. It builds on a successful pilot study.

3. It is multicentre, including secondary and tertiary units, reflecting current UK practice.

4. As a limitation, this study only includes women who have had a recent negative laparoscopy and therefore does not address whether gabapentin is useful in the management of chronic pelvic pain prior to surgical investigation or in those women where pathology is identified.

For peer review only

INTRODUCTION

Chronic pelvic pain (CPP) is as common as asthma, migraine and back pain[1], affecting more than 1 million women in the UK[2 3]. It is associated with significantly reduced quality of life (QoL)[4 5], a 45% reduction in work productivity and it has been estimated that caring for women with CPP in the UK costs £158 million annually[6 7]. CPP can be associated with underlying pathology such as endometriosis, but in up to 55% of women no obvious cause can be identified at laparoscopy[6]. Management of CPP is difficult when no pathology is identified, as no established gynaecological treatments are available. Due to its effectiveness in other chronic pain conditions, gabapentin (a GABA analogue), is increasingly being prescribed for CPP in both primary and secondary care[8]. However, there is no good quality evidence in CPP specifically on which to base this practice. To our knowledge, there is only one study evaluating the use of gabapentin for CPP, which did not have a placebo arm[9]. This small study in 56 women, compared gabapentin to amitriptyline and showed gabapentin to have greater efficacy at improving pain scores at 12 months. However, efficacy of gabapentin has been proven in other chronic pain conditions. A recent high quality review showed the number needed to treat (NNT) to be 5.8 (95% CI 4.3 to 9.0) to achieve at least 50% pain intensity reduction in painful diabetic neuropathy (829 patients); 7.5 (95% CI 5.2 to 14) to achieve at least 50% pain intensity reduction in postherpetic neuralgia (892 patients); and 5.4 (95% CI 2.9 to 31) to achieve at least 30% pain intensity reduction in fibromyalgia (150 patients)[8]. Moreover it is a drug that is very well tolerated: all-cause withdrawal rates are similar to placebo (gabapentin: 20%; placebo: 19%; number of studies: 17; number of participants: 3063)[8].

For peer review only - http://bmjopen.bmj.com/site/about/guidelines.xhtml

Given the clinical need for a medical treatment for CPP with no identifiable underlying pathology and the strong evidence supporting the acceptability and efficacy of gabapentin in other chronic pain conditions, we considered further investigation of gabapentin as a potential treatment for CPP in women was warranted. We hypothesise that treatment of women with CPP in the absence of any obvious pelvic pathology with gabapentin will alleviate pain and improve physical and emotional functioning. We initially performed a successful pilot randomised controlled trial (RCT) (GaPP1)[10 11]. Here we describe the protocol for our definitive multicentre trial to assess the efficacy of gabapentin in the management of CPP in women (GaPP2).

Objectives

Primary objective

The primary objective is to test the hypothesis that treatment with gabapentin has the potential to provide a safe, effective and convenient oral treatment and to prove if it can alleviate pain in women with CPP in the absence of any obvious pelvic pathology.

Secondary objective

The secondary objective is to test the hypothesis that treatment with gabapentin has the potential to improve physical and emotional functioning in women with CPP in the absence of any obvious pelvic pathology.

Outcomes

Primary outcome

We will employ co-primary outcome measures of average and worst pain scores recorded on a numerical rating scale (NRS). To capture the cyclicity that may occur

For peer review only - http://bmjopen.bmj.com/site/about/guidelines.xhtml

with CPP, weekly pain scores (on a 0-10 scale) will be recorded during the final four weeks of treatment (weeks 13-16 post randomisation), in the form of:

i) 'average pain this week' and

ii) 'worst pain this week'

Average pain score will be taken as the average of (i) and worst pain score as the worst response from (ii).

Secondary outcomes

- Physical and emotional function and quality of life
- Satisfaction with treatment
- Patient estimate of whether on active treatment or on placebo group, and confidence in and reasons for estimate
- Adherence to trial treatments, as reported by the participants
- Concomitant analgesic use, as reported by the participants
- Adverse events, as reported by participants (principally those that are serious and detailed in the summary of product characteristics and those that are unexpected)
- GP/hospital consultations, as reported by the participants
- Time off work and 'presenteeism'

METHODS AND ANALYSIS

Study Design

GaPP2 is a double blind placebo controlled randomised multi-centre clinical trial (figure 1). We will screen women with CPP from more than 8 NHS hospitals within the United Kingdom. Women will return weekly NRS pain scores to the trials office

For peer review only - http://bmjopen.bmj.com/site/about/guidelines.xhtml

for 4 weeks after initial consent. Those women meeting the inclusion criteria at the end of these 4 weeks will be randomised. We will randomise 300 women (150 to gabapentin, 150 to placebo). After randomisation and titration, participants will receive treatment with the maximum tolerated dose for 12 weeks. Participants and the healthcare team will be unblinded at the end of their treatment.

Participants

A total of 300 women with a history of chronic pelvic pain with no obvious pelvic pathology detected at laparoscopy will be recruited to the trial.

Inclusion criteria

- Women aged between 18-50 years
- CPP (non-cyclical with or without dysmenorrhoea or dyspareunia) of >3 months duration
- Pain located within the true pelvis or between and below anterior iliac crests
- No obvious pelvic pathology at laparoscopy (laparoscopy must have taken place at least 2 weeks ago, but no more than 36 months prior to screening)
- Using or willing to use effective contraception if necessary to avoid pregnancy
- Able to give informed consent
- For both the worst and average pre-randomisation NRS questions, at least three of the four weekly scores returned to the trials office. At least two of the worst pain scores should be ≥4/10.

Exclusion criteria

- Known pelvic pathology:
  - Endometriosis (macroscopic lesions)

For peer review only - http://bmjopen.bmj.com/site/about/guidelines.xhtml

  - complex or >5cm ovarian cyst
  - fibroid >3cm
  - dense adhesions
- Current malignancy under treatment
- Current use of gabapentin/pregabalin.
- Taking GnRH agonists and unable/unwilling to stop
- Surgery planned in the next 6 months
- History of significant renal impairment
- Previous reaction to gabapentin
- Breast feeding
- Pregnant
- Planning pregnancy in next 6 months
- Pain suspected to be of gastrointestinal origin (positive Rome III Diagnostic Criteria)
- Co-enrolment in another CTIMP

Participant enrolment

Research nurses (dedicated or through the National Institute for Health Research's Clinical Research Network, depending on the site) will be employed for the duration of the study to approach eligible women, provide them with patient information sheets and offer them the opportunity to discuss the trial, and obtain informed consent for screening. Consent will only be taken once the patient has had ample time to read the patient information sheet and had her questions answered.

For peer review only - http://bmjopen.bmj.com/site/about/guidelines.xhtml

Study settings

We will recruit patients from gynaecology outpatient clinics, gynaecology wards and day surgery units and chronic pelvic pain clinics within the UK.

Intervention and randomisation

Randomisation to gabapentin or placebo will occur once written informed consent has been obtained, final eligibility established from the pain responses provided during the screening phase, and baseline questionnaires completed. The Birmingham Clinical Trials Unit (BCTU) will provide third party web-based randomisation with telephone back-up. A minimisation procedure using a computer based algorithm will be used to avoid chance imbalances in treatment allocation and the following potentially important variables:

1. Presence or absence of dysmenorrhoea (a pain score of ≥4/10 will be considered significant)
2. Psychological distress measured by the General Health Questionnaire (scored as 0-12 with a cut off of 0-1 and 2-12 for minimisation)
3. Use of sex hormonal treatments (combined oral contraceptive, progestogens, levonorgestrel-releasing intrauterine system (Mirena®)).
4. Centre

A 'random element' will be included in the minimisation algorithm, so that each patient has a probability (unspecified here), of being randomised to the opposite treatment that they would have otherwise received. Full details of the algorithm used will be stored in a confidential document at BCTU. Both participants and the research team will remain blind to allocation.

For peer review only - http://bmjopen.bmj.com/site/about/guidelines.xhtml

Dose regimen

After randomisation, participants will be allocated a treatment pack containing either gabapentin or placebo oral tablets, both of identical appearance. Participants will start on 1 capsule (300mgs) daily and will increase by 1 capsule (300 mgs) increments every three days until they perceive that they are gaining adequate pain relief, or report side effects (eg dizziness, somnolence, mood changes, appetite and poor concentration) that preclude them from further increases, up to a maximum dose of 9 capsules (2700 mgs), as shown in Table 1. The titration phase will last a maximum of 4 weeks. If necessary they will be advised to titrate down to the last tolerated dose with minimal side effects. They will be asked to maintain their best tolerated dose until the end of week 16. Patients will be advised and given written instructions regarding their dosing regimen by a member of the research team. It will be recommended that the drug should be taken in three equally divided doses daily. The same protocol will be used for the placebo. When the participant stops treatment the dose will be reduced according to a dose reduction chart and written instructions will be given. Patients will be allowed to use other medication (including analgesics, self-medication and alternative treatments, e.g. acupuncture) throughout the study period.

Data collection

Data storage

All the data generated from the study will be stored in an in a bespoke database, which will be password protected. All paperwork will be kept in a locked filing cabinet in a locked office. All data will be stored in accordance with the Data Protection Act.

For peer review only - http://bmjopen.bmj.com/site/about/guidelines.xhtml

Screening

A member of the research team will assess the woman for eligibility to enter the screening phase. All data will be recorded on a CRF and transferred to a secure database, which will trigger the start of the weekly collection of pain scores.

Participant log

The clinical research team will keep an anonymised electronic log of women who fulfil the eligibility criteria, women who are invited to participate in the study, women recruited and women who leave the trial early. Reasons for non-recruitment (e.g. non-eligibility, refusal to participate, administrative error) will also be recorded. During the course of the study, we will document reasons for withdrawal from the study and loss to follow-up if available.

Pain scores

Pain NRS will be collected by an automated text messaging system. Two texts will be sent to the women's mobile phone, asking about average and worst pain respectively, and the woman will be asked to reply to the text message with her pain score, rating it from zero for no pain at all, to 10 being worst pain imaginable. To capture cyclicity, these will be collected weekly during the eligibility phase (weeks -1 – -4) and during the last 4 weeks of the treatment phase (weeks 13 – 16).

Treatment Diaries

Participants will be provided with a treatment diary at the same time as their medication pack is dispensed. The following measures will be completed by the participant daily from day 1 of treatment until week 16:

- Dose of gabapentin taken
- Reason for any change in trial medication dose
- Alternative therapies used

For peer review only

- Any visits to a healthcare professional

Questionnaires

A questionnaire will be given to all participants before randomisation but after screening (Baseline) and at 16 weeks post randomisation (See Table 2 for full schedule of assessments). This will include the following validated tools:

- 12-Item Short-Form Health Survey (SF-12): a quality of life measure[12].
- Brief Pain Inventory (BPI)[13]: a tool to measure pain intensity and interference of pain in a patient's life.
- Brief Fatigue Inventory (BFI)[14].
- General Health Questionnaire (GHQ)[15]: to identify psychological distress.
- Work and Productivity Activity Impairment (WPAI)[16].
- Pain catastrophising scale (PCS)[17].
- Sexual Activity Questionnaire (SAQ)[18].
- PainDETECT™: to identify a neuropathic component to pain[19].
- Pelvic Pain and Urinary/ Frequency Patient Symptom Scale (PUF) (at baseline only)

The questionnaire at baseline will include questions to capture the baseline demographic and clinical characteristics of the participants.

All questionnaires will be anonymised and completed in private.

Treatment diaries

The number of attendances to healthcare professionals for CPP and the use of concomitant medications will be collected using treatment diaries completed by the participants as necessary throughout the course of the trial.

For peer review only - http://bmjopen.bmj.com/site/about/guidelines.xhtml

Adverse Events

Participants will collect information about adverse events in their treatment diaries. However, they will be instructed to contact the clinical research team at any time after consenting to join the trial if they have an event that requires hospitalisation or an event that results in persistent or significant disability or incapacity. Gabapentin is generally well tolerated in the management of other chronic pain conditions, and serious adverse events are not anticipated. Any serious adverse events that occur after joining the trial will be reported in detail in the participant's medical notes, followed up until resolution of the event and reported to the ACCORD Research Governance (http://www.accord.ed.ac.uk) and QA Office based at the University of Edinburgh immediately or within 24 hours. ACCORD will onward report all SAEs to BCTU within 7 days.

Termination of Study

Participants will be unblinded at the end of the study and if taking gabapentin will have the option to continue on treatment or will be tapered off treatment. Participants who have been on placebo will be given the choice to start on gabapentin, which will be prescribed by their clinician.

Participants will be given an emergency contact card to carry while participating in the study. The blinding code will only be broken in emergency situations for reasons of patient safety, where knowledge of the treatment administered is necessary for the treatment of a serious adverse event. Participants whose randomisation codes are broken will cease treatment with the study drug, but will continue to be followed up. Participants may discontinue from the trial at any time at their own request, or they may be withdrawn at any time at the discretion of the research team for safety, behavioural or administrative reasons.

For peer review only - http://bmjopen.bmj.com/site/about/guidelines.xhtml

Data collection is envisaged to be complete in September 2018.

Sample size

We have based our sample size on being able to detect a minimally important difference (MID) in NRS scores with high levels of power. Studies have shown the MID in this population to be around 1 point on a 0-10 scale [20]. Our pilot study showed worst and average pain scores to have standard deviations between 2.0 and 2.5. If the SD is at the lower end of these estimates, 86 patients in each group (172 in total) would be required to have 90% power (p=0.05) to detect a difference of 1 point. If the SD is at the higher end, we could detect the same difference with 80% power (p=0.05) with 100 patients in each group. We have assumed the latter SD (2.5) to be conservative. To account for any increase in the risk of type I error that may be associated with having co-primary outcome measures we have applied a Bonferroni correction (alpha reduced to 0.025 from 0.05), which increases the sample size to 120 per group. Furthermore, to account for an expected average 20% loss to follow-up we will randomise 150 per group, 300 patients in total.

Proposed Analyses

Data analysis will be by intention to treat. Every attempt will be made to gather data on all women randomised, irrespective of compliance with the treatment protocol. Appropriate baseline characteristics, split by treatment group, will be presented for each outcome. Point estimates, 95% confidence intervals and p-values from two-sided tests will be reported.

Primary analysis

We will use a linear regression model to estimate differences in worst and average NRS scores between the two treatment groups, including baseline score and the

For peer review only - http://bmjopen.bmj.com/site/about/guidelines.xhtml

minimisation variables as covariates. The p-value from the associated chi-squared test will be produced and used to determine statistical significance. A Bonferroni correction will be applied as there are two primary outcomes. Further analysis using a repeated measures (multi-level) model will also be performed incorporating all eight recorded scores.

Secondary analysis

Data from the other continuous measures (SF-12, BPI, PCQ, SAQ, WPAIQ, BFI, PainDETECT™ and GHQ) will be analysed in a similar manner to the primary measure. Other outcome measures (use of permitted analgesic medication, satisfaction) will be analysed using standard methods (tests for trend, absolute/relative risks). Further analysis on pain scores will include an examination of the proportion of women that have a 30% and a 50% reduction in average and worst score from baseline as the outcome. A log-binomial regression model will be used here to generate adjusted relative risks.

Sub-group analyses will be limited to the same variables that were used as minimisation variables. Tests for statistical heterogeneity (e.g. by including treatment group by subgroup interaction parameter in the linear regression model) will be performed prior to any examination of effect estimate within subgroups.

Missing data and sensitivity analyses

Every attempt will be used to collect full follow up data on all women. In particular, participants will continue to be followed up even after protocol treatment violation. It is thus anticipated that missing data will be minimal. Patients with completely missing primary outcome data or with only one of four pain scores recorded will not be included in the primary analysis. Secondary sensitivity analyses will be performed to investigate the impact of missing data for the primary outcome: this will include a

For peer review only - http://bmjopen.bmj.com/site/about/guidelines.xhtml

worst score assumption. We will also simulate missing responses using a multiple imputation approach.

Trial Management

Professor Andrew Horne, Edinburgh, is the Chief Investigator of GaPP2. Mrs Ann Doust, University of Edinburgh is the Trial Manager (Tel: 0131 242 9492) and Ms Afia Sajid, Birmingham Clinical Trials Unit is the Trial Co-ordinator (Tel: 0121 414 8429). The trial will be coordinated by a Trial Management Group, comprising the grant holders, the Trial Manager and the Trial Co-ordinator. The Trial Office at the University of Birmingham Clinical Trials Unit (BCTU) is responsible for the day-to-day management. A Trial Steering Committee will oversee the conduct and progress of the trial and an independent Data Monitoring Committee will oversee the safety of participants within the trial. The grant holders are responsible for the design of the study, interpretation of data, writing of reports and decisions to submit reports for presentation or publication.

ETHICS AND DISSEMINATION

Ethical approval has been obtained from the Coventry and Warwick Research Ethics Committee (REC 15/WM/0036). The trial is registered with EudraCT (2014-005035-13) and ISRCTN (ISRCTN77451762). The University of Edinburgh and NHS Lothian are co-sponsors. All protocol amendments will be approved by the Chief Investigator and submitted in writing to the REC, Regulatory Authority and all local R&D departments. They will be communicated directly to all local investigating teams. Data will be presented at international conferences and published in peer-reviewed journals. We will make the information obtained from the study available to the public through relevant national bodies and charities.

For peer review only - http://bmjopen.bmj.com/site/about/guidelines.xhtml

DISCUSSION

CPP is a major public health issue for women throughout the developed world[2]. As with other chronic pain conditions it is associated with a marked reduction in quality of life and significant financial costs for the woman, her family and society as a whole[4 5]. When CPP is associated with underlying pathology such as endometriosis, therapies targeting the pathology can be initiated. However, in more than 50% of women no underlying cause is identified[6]. For these women, not only is it difficult to comprehend and come to terms with how there can be no associated pathology[21], there are also no available evidence-based treatments to consider.

The efficacy of a number of pharmacological and interventional therapies has been investigated for other chronic pain syndromes. There is increasing evidence that women with CPP demonstrate central changes similar to those associated with other forms of chronic pain[22 23] and thus it is likely that such therapies would also be effective for CPP. Moreover, recent work demonstrates a neuropathic component in a significant proportion of women with CPP[24], further supporting the investigation of drugs currently recommended for neuropathic pain[25] in women with CPP. The multicentre placebo-controlled RCT described here aims to contribute to the evidence base by assessing the efficacy of gabapentin in women with CPP with no underlying pathology. This trial is designed in line with the IMMPACT recommendations for the design of trials in chronic pain conditions[20 26 27] and builds upon a successful pilot study[10 11]. Women with CPP were surveyed to identify whether reduction in average or worst pain was most important to them. As there was no clear consensus (average 43.4%, worst 56.6%) co-primary outcomes of average and worst pain scores have

For peer review only - http://bmjopen.bmj.com/site/about/guidelines.xhtml

been chosen. We envisage the findings being of relevance to both primary and secondary care clinicians managing women with CPP.

REFERENCES

1. Zondervan KT, Yudkin PL, Vessey MP, et al. Prevalence and incidence of chronic pelvic pain in primary care: evidence from a national general practice database. BJOG 1999;**106**(11):1149-55.
2. Latthe P, Latthe M, Say L, et al. WHO systematic review of prevalence of chronic pelvic pain: a neglected reproductive health morbidity. BMC Public Health 2006;**6**:177.
3. Donaldson L. 150 years of the Annual Report of the Chief Medical Officer: On the state of public health 2008. London: DoH, 2009.
4. Breivik H, Collett B, Ventafridda V, et al. Survey of chronic pain in Europe: prevalence, impact on daily life, and treatment. Eur J Pain 2006;**10**(4):287-333.
5. Ayorinde AA, Macfarlane GJ, Saraswat L, et al. Chronic pelvic pain in women: an epidemiological perspective. Womens Health (Lond Engl) 2015;**11**(6):851-64.
6. Daniels JP, Khan KS. Chronic pelvic pain in women. BMJ 2010;**341**:c4834.
7. Cheong Y, William Stones R. Chronic pelvic pain: aetiology and therapy. Best Pract Res Clin Obstet Gynaecol 2006;**20**(5):695-711.
8. Wiffen PJ, Derry S, Moore RA, et al. Antiepileptic drugs for neuropathic pain and fibromyalgia - an overview of Cochrane reviews. Cochrane Database Syst Rev 2013;**11**:CD010567.
9. Sator-Katzenschlager SM, Scharbert G, Kress HG, et al. Chronic pelvic pain treated with gabapentin and amitriptyline: a randomized controlled pilot study. Wiener Klinische Wochenschrift 2005;**117**(21-22):761-8.
10. Horne AW, Critchley HO, Doust A, et al. GaPP: a pilot randomised controlled trial of the efficacy of action of gabapentin for the management of chronic pelvic pain in women: study protocol. BMJ Open 2012;**2**(3).
11. Lewis SC, Bhattacharya S, Wu O, et al. Gabapentin for the Management of Chronic Pelvic Pain in Women (GaPP1): A Pilot Randomised Controlled Trial. PLoS One 2016;**11**(4):e0153037.
12. Ware J, Jr., Kosinski M, Keller SD. A 12-Item Short-Form Health Survey: construction of scales and preliminary tests of reliability and validity. Med Care 1996;**34**(3):220-33.
13. Cleeland CS, Ryan KM. Pain assessment: global use of the Brief Pain Inventory. Ann Acad Med Singapore 1994;**23**(2):129-38.
14. Mendoza TR, Wang XS, Cleeland CS, et al. The rapid assessment of fatigue severity in cancer patients: use of the Brief Fatigue Inventory. Cancer 1999;**85**(5):1186-96.
15. Goldberg DP. *Manual of the General Health Questionnaire*. Windsor, England: NFER Publishing, 1978.
16. Reilly MC, Zbrozek AS, Dukes EM. The validity and reproducibility of a work productivity and activity impairment instrument. Pharmacoeconomics 1993;**4**(5):353-65.

For peer review only - http://bmjopen.bmj.com/site/about/guidelines.xhtml

17. Sullivan MJL, Bishop SR, Pivik J. The Pain Catastrophizing Scale: Development and validation. Psychol Assess 1995;**7**(4):524-32
18. Thirlaway K, Fallowfield L, Cuzick J. The Sexual Activity Questionnaire: a measure of women's sexual functioning. Qual Life Res 1996;**5**(1):81-90.
19. Freynhagen R, Baron R, Gockel U, et al. PainDETECT: a new screening questionnaire to identify neuropathic components in patients with back pain. Curr Med Res Opin 2006;**22**(10):1911-20.
20. Dworkin RH, Turk DC, Wyrwich KW, et al. Interpreting the clinical importance of treatment outcomes in chronic pain clinical trials: IMMPACT recommendations. J Pain 2008;**9**(2):105-21.
21. Grace VM. Problems women patients experience in the medical encounter for chronic pelvic pain: a New Zealand study. Health Care Women Int 1995;**16**(6):509-19.
22. Brawn J, Morotti M, Zondervan KT, et al. Central changes associated with chronic pelvic pain and endometriosis. Hum Reprod Update 2014;**20**(5):737-47.
23. Kaya S, Hermans L, Willems T, et al. Central sensitization in urogynecological chronic pelvic pain: a systematic literature review. Pain Physician 2013;**16**(4):291-308.
24. Whitaker LH, Reid J, Choa A, et al. An Exploratory Study into Objective and Reported Characteristics of Neuropathic Pain in Women with Chronic Pelvic Pain. PLoS One 2016;**11**(4):e0151950.
25. NICE. Neuropathic pain in adults: pharmacological management in non-specialist settings. NICE Clinical Guideline, 2013.
26. Dworkin RH, Turk DC, Farrar JT, et al. Core outcome measures for chronic pain clinical trials: IMMPACT recommendations. Pain 2005;**113**(1-2):9-19.
27. Dworkin RH, Turk DC, Peirce-Sandner S, et al. Research design considerations for confirmatory chronic pain clinical trials: IMMPACT recommendations. Pain 2010;**149**(2):177-93.

Authors' contributions

KV: research, contribution of original material, drafting, editing and approval of final manuscript; AH, SB, RC, JD, LM, WS: research, contribution of original material, editing and approval of final manuscript; AB, IT, JB, YC, BC, GM, AW: contribution of original material, editing and approval of final manuscript.

For peer review only

For peer review only - http://bmjopen.bmj.com/site/about/guidelines.xhtml

Funding statement

GaPP2 is funded by a grant from the NIHR/MRC Efficacy and Mechanism Evaluation (EME) Programme, Reference: 13/52/04.

Competing interests

KV has received research funding from Pfizer pharmaceuticals and Bayer Healthcare, honoraria from Bayer Healthcare, and consultancy fees from Grunenthal Gmb. APB has no competing issues as no direct funding from industry. APB is currently President of the British pain Society. SB receives grant funding from NIHR and Chief Scientist Office Scotland. He has been a speaker at a number of conferences that have received funding from the pharmaceutical industry. His clinical colleagues receive industry support for travel and for Departmental Seminars. JB declares no competing interests. YC receives grant funding from NIHR. RC has secured research funding from Allergan and is a member of Allergan's scientific advisory panel. He also receives grant funding from the NIHR via UCL Biomedical Research Centre. JD has nothing to declare. CAH declares no competing interests. AWH receives grant funding from the NIHR and the Medical Research Council. GJM is Chief Investigator of the British Society for Rheumatology (BSR) Biologics Register in Ankylosing Spondylitis. This is funded by the BSR who receive funds from Pfizer, AbbVie and UCB. LM declares no competing interests. WS declares no competing interests. IT has received honoraria and performs ad hoc consultancy from/to: Pfizer, Amgen and Lilly. She has academic collaborations and research contract support from: Abide Therapeutics and Grunenthal. AW has received a consulting fee from Astellas.

For peer review only - http://bmjopen.bmj.com/site/about/guidelines.xhtml

For peer review only

For peer review only - http://bmjopen.bmj.com/site/about/guidelines.xhtml

Figure 1: Study Flow chart

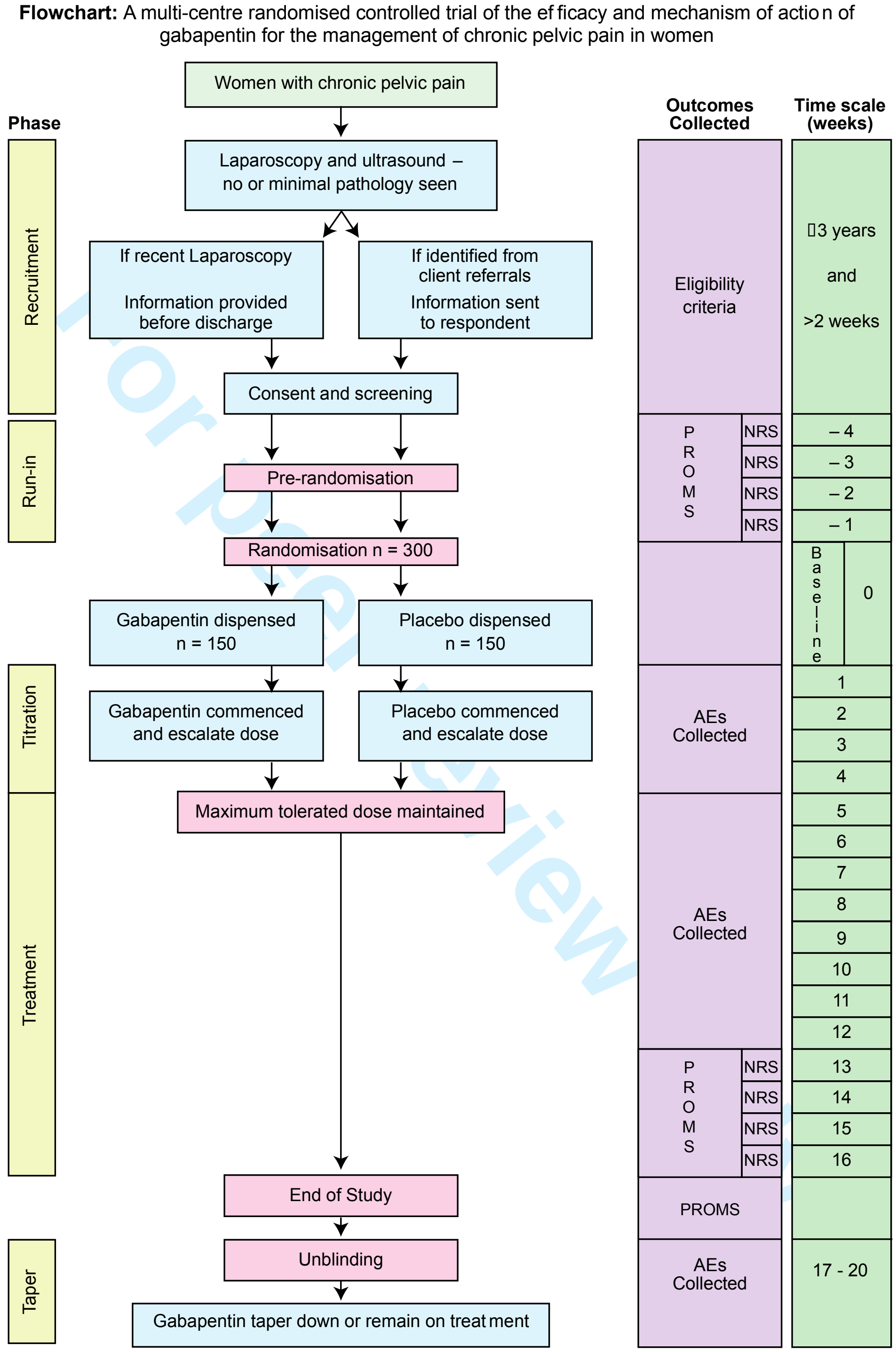

For peer review only - http://bmjopen.bmj.com/site/about/guidelines.xhtml

Table 1: Dose escalation schedule for GaPP2

Day in study	Total number of capsules/day (maximum)	Dosing	Maximum daily dose of gabapentin
1	1	1 capsule night	300 mg
2	1	1 capsule night	300 mg
3	1	1 capsule night	300 mg
4	2	1 capsule twice daily	600 mg
5	2	1 capsule twice daily	600 mg
6	2	1 capsule twice daily	600 mg
7	3	1 capsule three times daily	900 mg
8	3	1 capsule three times daily	900 mg
9	3	1 capsule three times daily	900 mg
10	4	1 capsule twice + 2 capsules at night	1200 mg
11	4	1 capsule twice + 2 capsules at night	1200 mg
12	4	1 capsule twice + 2 capsules at night	1200 mg
13	5	2 capsules twice + 1 capsule once	1500 mg
14	5	2 capsules twice + 1 capsule once	1500 mg
15	5	2 capsules twice + 1 capsule once	1500 mg
16	6	2 capsules three times daily	1800 mg
17	6	2 capsules three times daily	1800 mg
18	6	2 capsules three times daily	1800 mg
19	7	2 capsules twice + 3 capsules night	2100 mg
20	7	2 capsules twice + 3 capsules night	2100 mg
21	7	2 capsules twice + 3 capsules night	2100 mg
22	8	3 capsules twice + 2 capsules once	2400 mg
23	8	3 capsules twice + 2 capsules once	2400 mg
24	8	3 capsules twice + 2 capsules once	2400 mg
25	9	3 capsules three times daily	2700 mg
26	9	3 capsules three times daily	2700 mg
27	9	3 capsules three times daily	2700 mg
28 - 112	Remain on maximum tolerate dose until the end of week 16. (not exceeding 2700mg or 9 capsules per day). Daily dose should be divided equally into 3 doses.		

For peer review only - http://bmjopen.bmj.com/site/about/guidelines.xhtml

Table 2: Schedule of outcome assessments for GaPP2

**Phase**	**Run-in**	**Baseline, randomisation & treatment dispensed**	**Titration**	**Treatment**		**End of study & unblinding**	**Taper**
**Duration (weeks)**	-4 to -1	0	1-4	5-12	13-16		17-19
Weekly worst and average NRS	x x x x				x x x x		
Saliva sample		X					
SF12		X				X	
BPI		X				X	
PCQ		X				X	
SAQ		X				X	
BFI		X				X	
GHQ-12		X				X	
WPAIQ		X				X	
PainDETECT™		X				X	
PUF		X					
Adverse events			X	X	X	X	X
Permitted / Concomitant medication	X		X	X	X		X
Adherence or discontinuation			X	X	X		X

For peer review only

For peer review only - http://bmjopen.bmj.com/site/about/guidelines.xhtml

SPIRIT

STANDARD PROTOCOL ITEMS: RECOMMENDATIONS FOR INTERVENTIONAL TRIALS

For peer review only

SPIRIT 2013 Checklist: Recommended items to address in a clinical trial protocol and related documents*

Section/item	ItemNo	Description	Addressed on page number
**Administrative information**			
Title	1	Descriptive title identifying the study design, population, interventions, and, if applicable, trial acronym	1
Trial registration	2a	Trial identifier and registry name. If not yet registered, name of intended registry	4
	2b	All items from the World Health Organization Trial Registration Data Set	4, 20, 17, 10, 8, 9, 7, 6
Protocol version	3	Date and version identifier	-
Funding	4	Sources and types of financial, material, and other support	20
Roles and responsibilities	5a	Names, affiliations, and roles of protocol contributors	1
	5b	Name and contact information for the trial sponsor	17
	5c	Role of study sponsor and funders, if any, in study design; collection, management, analysis, and interpretation of data; writing of the report; and the decision to submit the report for publication, including whether they will have ultimate authority over any of these activities	17
	5d	Composition, roles, and responsibilities of the coordinating centre, steering committee, endpoint adjudication committee, data management team, and other individuals or groups overseeing the trial, if applicable (see Item 21a for data monitoring committee)	17

For peer review only - http://bmjopen.bmj.com/site/about/guidelines.xhtml

**Introduction**			
Background and rationale	6a	Description of research question and justification for undertaking the trial, including summary of relevant studies (published and unpublished) examining benefits and harms for each intervention	5
	6b	Explanation for choice of comparators	11
Objectives	7	Specific objectives or hypotheses	6
Trial design	8	Description of trial design including type of trial (eg, parallel group, crossover, factorial, single group), allocation ratio, and framework (eg, superiority, equivalence, noninferiority, exploratory)	7-8
**Methods: Participants, interventions, and outcomes**			
Study setting	9	Description of study settings (eg, community clinic, academic hospital) and list of countries where data will be collected. Reference to where list of study sites can be obtained	10
Eligibility criteria	10	Inclusion and exclusion criteria for participants. If applicable, eligibility criteria for study centres and individuals who will perform the interventions (eg, surgeons, psychotherapists)	8-9
Interventions	11a	Interventions for each group with sufficient detail to allow replication, including how and when they will be administered	10
	11b	Criteria for discontinuing or modifying allocated interventions for a given trial participant (eg, drug dose change in response to harms, participant request, or improving/worsening disease)	14
	11c	Strategies to improve adherence to intervention protocols, and any procedures for monitoring adherence (eg, drug tablet return, laboratory tests)	12
	11d	Relevant concomitant care and interventions that are permitted or prohibited during the trial	10

For peer review only - http://bmjopen.bmj.com/site/about/guidelines.xhtml

Outcomes	12	Primary, secondary, and other outcomes, including the specific measurement variable (eg, systolic blood pressure), analysis metric (eg, change from baseline, final value, time to event), method of aggregation (eg, median, proportion), and time point for each outcome. Explanation of the clinical relevance of chosen efficacy and harm outcomes is strongly recommended	6-7
Participant timeline	13	Time schedule of enrolment, interventions (including any run-ins and washouts), assessments, and visits for participants. A schematic diagram is highly recommended (see Figure)	23, 25
Sample size	14	Estimated number of participants needed to achieve study objectives and how it was determined, including clinical and statistical assumptions supporting any sample size calculations	15
Recruitment	15	Strategies for achieving adequate participant enrolment to reach target sample size	9
**Methods: Assignment of interventions (for controlled trials)**			
Allocation:			
Sequence generation	16a	Method of generating the allocation sequence (eg, computer-generated random numbers), and list of any factors for stratification. To reduce predictability of a random sequence, details of any planned restriction (eg, blocking) should be provided in a separate document that is unavailable to those who enrol participants or assign interventions	10
Allocation concealment mechanism	16b	Mechanism of implementing the allocation sequence (eg, central telephone; sequentially numbered, opaque, sealed envelopes), describing any steps to conceal the sequence until interventions are assigned	10
Implementation	16c	Who will generate the allocation sequence, who will enrol participants, and who will assign participants to interventions	10
Blinding (masking)	17a	Who will be blinded after assignment to interventions (eg, trial participants, care providers, outcome assessors, data analysts), and how	10

For peer review only - http://bmjopen.bmj.com/site/about/guidelines.xhtml

	17b	If blinded, circumstances under which unblinding is permissible, and procedure for revealing a participant's allocated intervention during the trial	14
**Methods: Data collection, management, and analysis**			
Data collection methods	18a	Plans for assessment and collection of outcome, baseline, and other trial data, including any related processes to promote data quality (eg, duplicate measurements, training of assessors) and a description of study instruments (eg, questionnaires, laboratory tests) along with their reliability and validity, if known. Reference to where data collection forms can be found, if not in the protocol	11-13, 25
	18b	Plans to promote participant retention and complete follow-up, including list of any outcome data to be collected for participants who discontinue or deviate from intervention protocols	14, 16
Data management	19	Plans for data entry, coding, security, and storage, including any related processes to promote data quality (eg, double data entry; range checks for data values). Reference to where details of data management procedures can be found, if not in the protocol	11
Statistical methods	20a	Statistical methods for analysing primary and secondary outcomes. Reference to where other details of the statistical analysis plan can be found, if not in the protocol	15-17
	20b	Methods for any additional analyses (eg, subgroup and adjusted analyses)	16
	20c	Definition of analysis population relating to protocol non-adherence (eg, as randomised analysis), and any statistical methods to handle missing data (eg, multiple imputation)	16
**Methods: Monitoring**			
Data monitoring	21a	Composition of data monitoring committee (DMC); summary of its role and reporting structure; statement of whether it is independent from the sponsor and competing interests; and reference to where further details about its charter can be found, if not in the protocol. Alternatively, an explanation of why a DMC is not needed	17

	21b	Description of any interim analyses and stopping guidelines, including who will have access to these interim results and make the final decision to terminate the trial	-
Harms	22	Plans for collecting, assessing, reporting, and managing solicited and spontaneously reported adverse events and other unintended effects of trial interventions or trial conduct	14
Auditing	23	Frequency and procedures for auditing trial conduct, if any, and whether the process will be independent from investigators and the sponsor	17
**Ethics and dissemination**			
Research ethics approval	24	Plans for seeking research ethics committee/institutional review board (REC/IRB) approval	17
Protocol amendments	25	Plans for communicating important protocol modifications (eg, changes to eligibility criteria, outcomes, analyses) to relevant parties (eg, investigators, REC/IRBs, trial participants, trial registries, journals, regulators)	17
Consent or assent	26a	Who will obtain informed consent or assent from potential trial participants or authorised surrogates, and how (see Item 32)	9
	26b	Additional consent provisions for collection and use of participant data and biological specimens in ancillary studies, if applicable	-
Confidentiality	27	How personal information about potential and enrolled participants will be collected, shared, and maintained in order to protect confidentiality before, during, and after the trial	11
Declaration of interests	28	Financial and other competing interests for principal investigators for the overall trial and each study site	21
Access to data	29	Statement of who will have access to the final trial dataset, and disclosure of contractual agreements that limit such access for investigators	17
Ancillary and post-trial care	30	Provisions, if any, for ancillary and post-trial care, and for compensation to those who suffer harm from trial participation	14

For peer review only - http://bmjopen.bmj.com/site/about/guidelines.xhtml

Dissemination policy	31a	Plans for investigators and sponsor to communicate trial results to participants, healthcare professionals, the public, and other relevant groups (eg, via publication, reporting in results databases, or other data sharing arrangements), including any publication restrictions	17
	31b	Authorship eligibility guidelines and any intended use of professional writers	17
	31c	Plans, if any, for granting public access to the full protocol, participant-level dataset, and statistical code	-
**Appendices**			
Informed consent materials	32	Model consent form and other related documentation given to participants and authorised surrogates	-
Biological specimens	33	Plans for collection, laboratory evaluation, and storage of biological specimens for genetic or molecular analysis in the current trial and for future use in ancillary studies, if applicable	-

*It is strongly recommended that this checklist be read in conjunction with the SPIRIT 2013 Explanation & Elaboration for important clarification on the items. Amendments to the protocol should be tracked and dated. The SPIRIT checklist is copyrighted by the SPIRIT Group under the Creative Commons "Attribution-NonCommercial-NoDerivs 3.0 Unported" license.

For peer review only - http://bmjopen.bmj.com/site/about/guidelines.xhtml

GaPP2: A multi-centre randomised controlled trial of the efficacy of gabapentin for the management of chronic pelvic pain in women: study protocol

Journal:	*BMJ Open*
Manuscript ID	bmjopen-2016-014924.R1
Article Type:	Protocol
Date Submitted by the Author:	11-Sep-2017
Complete List of Authors:	Vincent, Katy; University of Oxford, Nuffield Department of Obstetrics and Gyanecology<br>Baranowski, Andrew ; The National Hospital for Neurology and Neurosurgery , Pain Management Centre<br>Bhattacharya, Siladitya; Aberdeen University, Obstetrics and Gynaecology<br>Birch, Judy; Pelvic Pain Support Network<br>Cheong, Ying; University of Southampton Faculty of Medicine<br>Cregg, Roman; The National Hospital for Neurology and Neurosurgery, Pain Management Centre<br>Daniels, Jane; University of Birmingham, Birmingham Clinical Trials Unit<br>Hewitt, Catherine; University of Birmingham, Clinical Trials Unit<br>Macfarlane, Gary; University of Aberdeen, Epidemiology Group<br>Middleton, Lee; University of Birmingham, Clinical Trials Unit<br>Szubert, Wojciech; University of Edinburgh, MRC Centre for Reproductive Health<br>Tracey, Irene; University of Oxford, Nuffield Department of Clinical Neurosciences<br>Williams, Amanda; University College London, Research Department of Clinical, Educational and Health Psychology<br>Horne, Andrew
**Primary Subject Heading**:	Obstetrics and gynaecology
Secondary Subject Heading:	Evidence based practice, Anaesthesia
Keywords:	gabapentin, chronic pelvic pain, Pain management < ANAESTHETICS

SCHOLARONE™
Manuscripts

TITLE PAGE

Title: GaPP2: A multi-centre randomised controlled trial of the efficacy of gabapentin for the management of chronic pelvic pain in women: study protocol

Authors: Katy Vincent[1], Andrew Baranowski[2], Siladitya Bhattacharya[3], Judy Birch[4], Ying Cheong[5], Roman Cregg[2], Jane Daniels[6], Catherine A. Hewitt[6], Gary J Macfarlane[7], Lee Middleton[6], Wojciech Szubert[8], Irene Tracey[9], Amanda C de C Williams[10], Andrew W Horne[8]

[1] Nuffield Department of Obstetrics and Gynaecology, University of Oxford, OX3 9DU, UK.

[2] Pain Management Centre, The National Hospital for Neurology and Neurosurgery, London W1T 4AJ, UK.

[3] Institute of Applied Health Sciences, School of Medicine, Medical Sciences and Nutrition, University of Aberdeen, UK

[4] Pelvic Pain Support Network, Poole, BH17 9AT, UK.

[5] Faculty of Medicine, University of Southampton, SO16 5YA, UK.

[6] Birmingham Clinical Trials Unit, University of Birmingham. B15 2TT. UK

[7] Epidemiology Group, School of Medicine, Medical Sciences and Nutrition, University of Aberdeen, UK

[8] MRC Centre for Reproductive Health, Queen's Medical Research Institute, 47 Little France Crescent, Edinburgh EH16 4TJ

[9] Nuffield Department of Clinical Neurosciences, University of Oxford, OX3 9DU, UK.

[10] Research Dept of Clinical, Educational & Health Psychology, University College London, Gower St, London WC1E 6BT

For peer review only - http://bmjopen.bmj.com/site/about/guidelines.xhtml

Corresponding author:

Professor Andrew Horne

MRC Centre for Reproductive Health

Queen's Medical Research Institute

47 Little France Crescent

Edinburgh EH16 4TJ

Tel: +44 131 242 6988

Email: andrew.horne@ed.ac.uk

Key Words: Chronic Pelvic Pain; Gabapentin; Randomised Controlled Trial

Word Count: 3380

For peer review only

ABSTRACT

Introduction:

Chronic pelvic pain (CPP) affects more than 1 million UK women with associated healthcare costs of £158 million annually. Current evidence supporting interventions when no underlying pathology is identified is very limited and treatment is frequently inadequate. Gabapentin (a GABA analogue) is efficacious and often well tolerated in other chronic pain conditions. We have completed a successful pilot randomised controlled trial (GaPP1) and here describe the protocol for the definitive multicentre trial to assess the efficacy of gabapentin in the management of CPP in women (GaPP2).

Methods and analysis:

We plan to perform a double blind placebo controlled randomised multi-centre clinical trial, recruiting 300 women with CPP from more than 8 NHS hospitals within the UK. After randomisation, women will titrate their medication (gabapentin or placebo) over a 4-week period to a maximum of 2700mg or placebo equivalent and will then maintain a stable dose for a 12 week period. Response to treatment will be monitored with validated questionnaires and co-primary outcome measures of average and worst pain scores will be employed. The primary objective is to test the hypothesis that treatment with gabapentin has the potential to provide a safe and effective oral treatment and whether it can alleviate pain in women with CPP in the absence of any obvious pelvic pathology.

Ethics and dissemination:

Ethical approval has been obtained from the Coventry and Warwick Research Ethics Committee (REC 15/WM/0036). Data will be presented at international conferences

For peer review only

For peer review only - http://bmjopen.bmj.com/site/about/guidelines.xhtml

and published in peer-reviewed journals. We will make the information obtained from the study available to the public through national bodies and charities.

Trial registration number: ISRCTN77451762

For peer review only

For peer review only - http://bmjopen.bmj.com/site/about/guidelines.xhtml

INTRODUCTION

Chronic pelvic pain (CPP) affects more than 1 million women in the UK[1-3]. It is associated with significantly reduced quality of life (QoL)[4 5], a 45% reduction in work productivity and it has been estimated that caring for women with CPP in the UK costs £158 million annually[6 7]. CPP can be associated with underlying pathology such as endometriosis, but in up to 55% of women no obvious cause can be identified at laparoscopy[6]. Management of CPP is difficult when no pathology is identified, as no established gynaecological treatments are available. Due to its effectiveness in other chronic pain conditions, gabapentin (a GABA analogue), is increasingly being prescribed for CPP in both primary and secondary care[8]. However, there is no good quality evidence in CPP specifically on which to base this practice[9]. To our knowledge, there is only one study evaluating the use of gabapentin for CPP, which did not have a placebo arm[10]. This small study in 56 women, compared gabapentin to amitriptyline and showed gabapentin to have greater efficacy at improving pain scores at 12 months. However, efficacy of gabapentin has been proven in other chronic pain conditions. A recent high quality review showed the number needed to treat (NNT) to be 5.8 (95% CI 4.3 to 9.0) to achieve at least 50% pain intensity reduction in painful diabetic neuropathy (829 patients); 7.5 (95% CI 5.2 to 14) to achieve at least 50% pain intensity reduction in postherpetic neuralgia (892 patients); and 5.4 (95% CI 2.9 to 31) to achieve at least 30% pain intensity reduction in fibromyalgia (150 patients)[8]. Moreover it is a drug that is very well tolerated: all-cause withdrawal rates are similar to placebo (gabapentin: 20%; placebo: 19%; number of studies: 17; number of participants: 3063)[8].

For peer review only - http://bmjopen.bmj.com/site/about/guidelines.xhtml

Given the clinical need for a medical treatment for CPP with no identifiable underlying pathology and the strong evidence supporting the acceptability and efficacy of gabapentin in other chronic pain conditions, we considered further investigation of gabapentin as a potential treatment for CPP in women was warranted. We hypothesise that treatment of women with CPP in the absence of any obvious pelvic pathology with gabapentin will alleviate pain and improve physical and emotional functioning. We initially performed a successful pilot randomised controlled trial (RCT) (GaPP1)[11 12]. Here we describe the protocol for our definitive multicentre trial to assess the efficacy of gabapentin in the management of CPP in women (GaPP2).

Objectives

Primary objective

The primary objective is to test the hypothesis that treatment with gabapentin has the potential to provide a safe and effective oral treatment and to prove if it can alleviate pain in women with CPP in the absence of any obvious pelvic pathology.

Secondary objective

The secondary objective is to test the hypothesis that treatment with gabapentin has the potential to improve physical and emotional functioning in women with CPP in the absence of any obvious pelvic pathology.

Outcomes

Primary outcome

We will employ co-primary outcome measures of average and worst pain scores recorded on a numerical rating scale (NRS). To capture the cyclicity that may occur

For peer review only - http://bmjopen.bmj.com/site/about/guidelines.xhtml

with CPP, weekly pain scores (on a 0-10 scale) will be recorded during the final four weeks of treatment (weeks 13-16 post randomisation), in the form of:

i) ‘average pain this week’ and

ii) ‘worst pain this week’

The composite ‘average’ pain score will be taken as the average of the four weekly average pain scores submitted, and the composite ‘worst’ pain score as the worst of the four weekly worst pain scores submitted.

Secondary outcomes

- Physical and emotional function and quality of life
- Satisfaction with treatment
- Patient estimate of whether on active treatment or on placebo group, and confidence in and reasons for estimate
- Adherence to trial treatments, as reported by the participants
- Concomitant analgesic use, as reported by the participants
- Adverse events, as reported by participants (principally those that are serious and detailed in the summary of product characteristics and those that are unexpected)
- GP/hospital consultations, as reported by the participants
- Time off work and ‘presenteeism’

METHODS AND ANALYSIS

Study Design

GaPP2 is a double blind placebo controlled randomised multi-centre clinical trial (Figure 1). We will screen women with CPP from more than 8 NHS hospitals within

For peer review only - http://bmjopen.bmj.com/site/about/guidelines.xhtml

the United Kingdom. Women will return weekly NRS pain scores to the trials office for 4 weeks after initial consent. Those women meeting the inclusion criteria at the end of these 4 weeks will be randomised. We will randomise 300 women (150 to gabapentin, 150 to placebo). After randomisation and titration, participants will receive treatment with the maximum tolerated dose for 12 weeks. Participants and the healthcare team will be unblinded at the end of their treatment.

Participants

A total of 300 women with a history of chronic pelvic pain with no obvious pelvic pathology detected at laparoscopy will be recruited to the trial.

Inclusion criteria

- Women aged between 18-50 years
- CPP (~~non-cyclical~~ with or without dysmenorrhoea or dyspareunia) of >3 months duration
- Pain located within the true pelvis or between and below anterior iliac crests
- No obvious pelvic pathology at laparoscopy (laparoscopy must have taken place at least 2 weeks ago, but no more than 36 months prior to screening)
- Using or willing to use effective contraception if necessary to avoid pregnancy
- Able to give informed consent
- For both the worst and average pre-randomisation NRS questions, at least three of the four weekly scores returned to the trials office. At least two of the worst pain scores should be ≥4/10. Potential participants who have been on a stable dose of an analgesic, other than gabapentin or pregabalin, for at least 4 weeks prior to screening will be eligible.

For peer review only - http://bmjopen.bmj.com/site/about/guidelines.xhtml

Exclusion criteria

- Dysmenorrhoea alone
- Known pelvic pathology:
  - Endometriosis (macroscopic lesions)
  - complex or >5cm ovarian cyst
  - fibroid >3cm
  - dense adhesions
- Current malignancy under treatment
- Current use of gabapentin/pregabalin.
- Taking GnRH agonists and unable/unwilling to stop
- Surgery planned in the next 6 months
- History of significant renal impairment
- Previous allergic reaction to gabapentin
- Breast feeding
- Pregnant
- Planning pregnancy in next 6 months
- Pain suspected to be of gastrointestinal origin (positive Rome III Diagnostic Criteria)
- Co-enrolment in another Clinical Trial of an Investigational Medicinal Product

Participant enrolment

Research nurses (dedicated or through the National Institute for Health Research's Clinical Research Network, depending on the site) will be employed for the duration of the study to approach eligible women, provide them with patient information sheets and offer them the opportunity to discuss the trial, and obtain informed consent for

For peer review only - http://bmjopen.bmj.com/site/about/guidelines.xhtml

screening. Consent will only be taken once the patient has had ample time to read the patient information sheet and had her questions answered.

Study settings

We will recruit patients from gynaecology outpatient clinics, gynaecology wards and day surgery units and chronic pelvic pain clinics within the UK.

Intervention and randomisation

Randomisation to gabapentin or placebo will occur once written informed consent has been obtained, final eligibility established from the pain responses provided during the screening phase, and baseline questionnaires completed. The Birmingham Clinical Trials Unit (BCTU) will provide third party web-based randomisation with telephone back-up. A minimisation procedure using a computer based algorithm will be used to avoid chance imbalances in treatment allocation and the following potentially important variables:

1. Presence or absence of dysmenorrhoea (a pain score of ≥4/10 will be considered significant)
2. Psychological distress measured by the General Health Questionnaire (scored as 0-12 with a cut off of 0-1 and 2-12 for minimisation)
3. Use of sex hormonal treatments (combined oral contraceptive, progestogens, levonorgestrel-releasing intrauterine system (Mirena®)).
4. Centre

A 'random element' will be included in the minimisation algorithm, so that each patient has a probability (unspecified here), of being randomised to the opposite treatment that they would have otherwise received. Full details of the algorithm used

For peer review only - http://bmjopen.bmj.com/site/about/guidelines.xhtml

will be stored in a confidential document at BCTU. Both participants and the research team will remain blind to allocation.

Dose regimen

After randomisation, participants will be allocated a trial treatment pack from the hospital pharmacy containing either gabapentin or placebo oral tablets, both of identical appearance. Participants will start on 1 capsule (300mgs) daily and will increase by 1 capsule (300 mgs) increments every three days until they perceive that they are gaining adequate pain relief, or report side effects (e.g. dizziness, somnolence, mood changes, appetite and poor concentration) that preclude them from further increases, up to a maximum dose of 9 capsules (2700 mgs), as shown in Table 1. The titration phase will last a maximum of 4 weeks. If necessary they will be advised to titrate down to the last tolerated dose with minimal side effects. They will be asked to maintain their best tolerated dose until the end of week 16. Patients will be advised and given written instructions regarding their dosing regimen by a member of the research team. It will be recommended that the drug should be taken in three equally divided doses daily. The same protocol will be used for the placebo. When the participant stops treatment the dose will be reduced according to a dose reduction chart and written instructions will be given. Patients will be allowed to use other medication (including analgesics, self-medication and alternative treatments, e.g. acupuncture) throughout the study period.

For peer review only - http://bmjopen.bmj.com/site/about/guidelines.xhtml

Data collection

Data storage

All the data generated from the study will be stored in a bespoke database, which will be password protected. All paperwork will be kept in a locked filing cabinet in a locked office. All data will be stored in accordance with the Data Protection Act.

Screening

A member of the research team will assess the woman for eligibility to enter the screening phase. All data will be recorded on a CRF and transferred to a secure database, which will trigger the start of the weekly collection of pain scores.

Participant log

The clinical research team will keep an anonymised electronic log of women who fulfil the eligibility criteria, women who are invited to participate in the study, women recruited and women who leave the trial early. Reasons for non-recruitment (e.g. non-eligibility, refusal to participate, administrative error) will also be recorded. During the course of the study, we will document reasons for withdrawal from the study and loss to follow-up if available.

Pain scores

Pain NRS will be collected by an automated text messaging system. Two texts will be sent to the women's mobile phone, asking about average and worst pain respectively, and the woman will be asked to reply to the text message with her pain score, rating it from zero for no pain at all, to 10 being worst pain imaginable. To capture cyclicity, these will be collected weekly during the eligibility phase (weeks -1 – -4) and during the last 4 weeks of the treatment phase (weeks 13 – 16).

For peer review only

For peer review only - http://bmjopen.bmj.com/site/about/guidelines.xhtml

Treatment Diaries

Participants will be provided with a treatment diary at the same time as their medication pack is dispensed. The following measures will be completed by the participant daily from day 1 of treatment until week 16:

- Dose of gabapentin taken
- Reason for any change in trial medication dose
- Alternative therapies used
- Any visits to a healthcare professional

Questionnaires

A questionnaire will be given to all participants before randomisation but after screening (Baseline) and at 16 weeks post randomisation (See Table 2 for full schedule of assessments). This will include the following validated tools:

- 12-Item Short-Form Health Survey (SF-12): a quality of life measure[13].
- Brief Pain Inventory (BPI)[14]: a tool to measure pain intensity and interference of pain in a patient's life.
- Brief Fatigue Inventory (BFI)[15].
- General Health Questionnaire (GHQ)[16]: to identify psychological distress.
- Work and Productivity Activity Impairment (WPAI)[17].
- Pain catastrophising scale (PCS)[18].
- Sexual Activity Questionnaire (SAQ)[19].
- PainDETECT™: to identify a neuropathic component to pain[20].
- Pelvic Pain and Urinary/ Frequency Patient Symptom Scale (PUF) (at baseline only)

The questionnaire at baseline will include questions to capture the baseline demographic and clinical characteristics of the participants.

For peer review only

For peer review only - http://bmjopen.bmj.com/site/about/guidelines.xhtml

All questionnaires will be anonymised and completed in private.

Adverse Events

Participants will collect information about adverse events in their treatment diaries. However, they will be instructed to contact the clinical research team at any time after consenting to join the trial if they have an event that requires hospitalisation or an event that results in persistent or significant disability or incapacity. Gabapentin is generally well tolerated in the management of other chronic pain conditions, and serious adverse events are not anticipated. Any serious adverse events that occur after joining the trial will be reported in detail in the participant's medical notes and followed up until resolution of the event. The assessment of seriousness, causality and expectedness will be conducted assuming that the participant received gabapentin, with the blinding not broken. All SAEs will be reported to the ACCORD Research Governance (http://www.accord.ed.ac.uk) and QA Office based at the University of Edinburgh immediately or within 24 hours. ACCORD will onward report all SAEs to BCTU within 7 days.

Termination of Study

Participants will be unblinded at the end of the study and if taking gabapentin will have the option to continue on treatment or will be tapered off treatment. Participants who have been on placebo will be given the choice to start on gabapentin, which will be prescribed by their clinician. Participants will be given an emergency contact card to carry while participating in the study. The blinding code will only be broken in emergency situations for reasons of patient safety, where knowledge of the treatment administered is necessary for the treatment of a serious adverse event. Participants whose randomisation codes are broken will cease treatment with the study drug, but will continue to be followed up. Participants may discontinue from the trial at any

For peer review only - http://bmjopen.bmj.com/site/about/guidelines.xhtml

time at their own request, or they may be withdrawn at any time at the discretion of the research team for safety, behavioural or administrative reasons. Data collection is envisaged to be complete in September 2018.

Sample size

We have based our sample size on being able to detect a minimally important difference (MID) in NRS scores with high levels of power. Studies have shown the MID in this population to be around 1 point on a 0-10 scale [20]. Our pilot study showed worst and average pain scores to have standard deviations between 2.0 and 2.5. If the SD is at the lower end of these estimates, 86 patients in each group (172 in total) would be required to have 90% power (p=0.05) to detect a difference of 1 point. If the SD is at the higher end, we could detect the same difference with 80% power (p=0.05) with 100 patients in each group. We have assumed the latter SD (2.5) to be conservative. To account for any increase in the risk of type I error that may be associated with having co-primary outcome measures we have applied a Bonferroni correction (alpha reduced to 0.025 from 0.05), which increases the sample size to 120 per group. Furthermore, to account for an expected average 20% loss to follow-up we will randomise 150 per group, 300 patients in total.

Proposed Analyses

Data analysis will be by intention to treat. Every attempt will be made to gather data on all women randomised, irrespective of compliance with the treatment protocol. Appropriate baseline characteristics, split by treatment group, will be presented for each outcome. Point estimates, 95% confidence intervals and p-values from two-sided tests will be reported.

For peer review only - http://bmjopen.bmj.com/site/about/guidelines.xhtml
For peer review only

Primary analysis

We will use a linear regression model to estimate differences in worst and average NRS scores between the two treatment groups, including baseline score and the minimisation variables as covariates. The p-value from the associated chi-squared test will be produced and used to determine statistical significance. A Bonferroni correction will be applied as there are two primary outcomes. Further analysis using a repeated measures (multi-level) model will also be performed incorporating all eight recorded scores.

Secondary analysis

Data from the other continuous measures (SF-12, BPI, PCQ, SAQ, WPAIQ, BFI, PainDETECT™ and GHQ) will be analysed in a similar manner to the primary measure. Other outcome measures (use of permitted analgesic medication, satisfaction) will be analysed using standard methods (tests for trend, absolute/relative risks). Further analysis on pain scores will include an examination of the proportion of women that have a 30% and a 50% reduction in average and worst score from baseline as the outcome. A log-binomial regression model will be used here to generate adjusted relative risks. Sub-group analyses will be limited to the same variables that were used as minimisation variables. Tests for statistical heterogeneity (e.g. by including treatment group by subgroup interaction parameter in the linear regression model) will be performed prior to any examination of effect estimate within subgroups. In addition, we will investigate up to nine clinical variables measured at baseline to determine whether they correlate with response to treatment. These will include the minimisation variables (presence of dysmenorrhoea/psychological distress/current use of hormonal treatment) along with measures of intensity and of nature of pain (e.g. PainDETECT™), number of

For peer review only - http://bmjopen.bmj.com/site/about/guidelines.xhtml

functional systems involved (as a measure of organ specific versus generalised pelvic pain syndrome) and PUF score.

Missing data and sensitivity analyses

Every attempt will be used to collect full follow up data on all women. In particular, participants will continue to be followed up even after protocol treatment violation. It is thus anticipated that missing data will be minimal. Patients with completely missing primary outcome data or with only one of four pain scores recorded will not be included in the primary analysis. Secondary sensitivity analyses will be performed to investigate the impact of missing data for the primary outcome: this will include a worst score assumption. We will also simulate missing responses using a multiple imputation approach.

ETHICS AND DISSEMINATION

Ethical approval has been obtained from the Coventry and Warwick Research Ethics Committee (REC 15/WM/0036). Data will be presented at international conferences and published in peer-reviewed journals. We will make the information obtained from the study available to the public through relevant national bodies and charities.

RESEARCH GOVERNANCE

We shall adopt the standard approach used for monitoring randomised controlled trials and have a Trial Steering Committee (TSC) of at least four independent members, including pain specialist, a gynaecologist, trial methodologist and a PPI representative. There will also be a Data Monitoring Committee (DMC) comprising three independent members (a pain specialist, a gynaecologist and a statistician with extensive trial experience) who will review interim analyses. The terms of reference

For peer review only - http://bmjopen.bmj.com/site/about/guidelines.xhtml

and charter for this DMC will be guided by the DAMOCLES project, and we anticipate the DMC and TSC will meet biannually.

DISCUSSION

CPP is a major public health issue for women throughout the developed world[2]. As with other chronic pain conditions it is associated with a marked reduction in quality of life and significant financial costs for the woman, her family and society as a whole[4 5]. When CPP is associated with underlying pathology such as endometriosis, therapies targeting the pathology can be initiated. However, in more than 50% of women no underlying cause is identified[6]. For these women, not only is it difficult to comprehend and come to terms with how there can be no associated pathology[22], there are also no available evidence-based treatments to consider.

The efficacy of a number of pharmacological and interventional therapies has been investigated for other chronic pain syndromes. There is increasing evidence that women with CPP demonstrate central changes similar to those associated with other forms of chronic pain[23 24] and thus it is likely that such therapies would also be effective for CPP. Moreover, recent work demonstrates a neuropathic component in a significant proportion of women with CPP[25], further supporting the investigation of drugs currently recommended for neuropathic pain[26] in women with CPP. The multicentre placebo-controlled RCT described here aims to contribute to the evidence base by assessing the efficacy of gabapentin in women with CPP with no underlying pathology[9]. This trial is designed in line with the IMMPACT recommendations for the design of trials in chronic pain conditions[21 27 28] and builds upon a successful pilot study[11 12]. Women with CPP were surveyed to identify whether reduction in average

For peer review only - http://bmjopen.bmj.com/site/about/guidelines.xhtml

or worst pain was most important to them. As there was no clear consensus (average 43.4%, worst 56.6%) co-primary outcomes of average and worst pain scores have been chosen. We envisage the findings being of relevance to both primary and secondary care clinicians managing women with CPP.

REFERENCES

1. Zondervan KT, Yudkin PL, Vessey MP, et al. Prevalence and incidence of chronic pelvic pain in primary care: evidence from a national general practice database. British Journal of Obstetrics and Gynaecology 1999;**106**(11):1149-55.
2. Latthe P, Latthe M, Say L, et al. WHO systematic review of prevalence of chronic pelvic pain: a neglected reproductive health morbidity. BMC Public Health 2006;**6**:177.
3. Donaldson L. 150 years of the Annual Report of the Chief Medical Officer: On the state of public health 2008. London: DoH, 2009.
4. Breivik H, Collett B, Ventafridda V, et al. Survey of chronic pain in Europe: prevalence, impact on daily life, and treatment. Eur J Pain 2006;**10**(4):287-333.
5. Ayorinde AA, Macfarlane GJ, Saraswat L, et al. Chronic pelvic pain in women: an epidemiological perspective. Womens Health (Lond Engl) 2015;**11**(6):851-64.
6. Daniels JP, Khan KS. Chronic pelvic pain in women. BMJ 2010;**341**:c4834.
7. Cheong Y, William Stones R. Chronic pelvic pain: aetiology and therapy. Best Pract Res Clin Obstet Gynaecol 2006;**20**(5):695-711.
8. Wiffen PJ, Derry S, Moore RA, et al. Antiepileptic drugs for neuropathic pain and fibromyalgia - an overview of Cochrane reviews. Cochrane Database Syst Rev 2013;**11**:CD010567.
9. Horne AW, Vincent K, Cregg, Daniels J. Is gabapentin effective for women with unexplained chronic pelvic pain? BMJ (in press - publication date 23 09 17)
10. Sator-Katzenschlager SM, Scharbert G, Kress HG, et al. Chronic pelvic pain treated with gabapentin and amitriptyline: a randomized controlled pilot study. Wiener klinische Wochenschrift 2005;**117**(21-22):761-8.
11. Horne AW, Critchley HO, Doust A, et al. GaPP: a pilot randomised controlled trial of the efficacy of action of gabapentin for the management of chronic pelvic pain in women: study protocol. BMJ Open 2012;**2**(3).
12. Lewis SC, Bhattacharya S, Wu O, et al. Gabapentin for the Management of Chronic Pelvic Pain in Women (GaPP1): A Pilot Randomised Controlled Trial. PLoS One 2016;**11**(4):e0153037.
13. Ware J, Jr., Kosinski M, Keller SD. A 12-Item Short-Form Health Survey: construction of scales and preliminary tests of reliability and validity. Med Care 1996;**34**(3):220-33.
14. Cleeland CS, Ryan KM. Pain assessment: global use of the Brief Pain Inventory. Annals of the Academy of Medicine, Singapore 1994;**23**(2):129-38.

For peer review only - http://bmjopen.bmj.com/site/about/guidelines.xhtml

15. Mendoza TR, Wang XS, Cleeland CS, et al. The rapid assessment of fatigue severity in cancer patients: use of the Brief Fatigue Inventory. Cancer 1999;**85**(5):1186-96.
16. Goldberg DP. *Manual of the General Health Questionnaire*. Windsor, England: NFER Publishing, 1978.
17. Reilly MC, Zbrozek AS, Dukes EM. The validity and reproducibility of a work productivity and activity impairment instrument. Pharmacoeconomics 1993;**4**(5):353-65.
18. Sullivan MJL, Bishop SR, Pivik J. The Pain Catastrophizing Scale: Development and validation. Psychol Assess 1995;**7**(4):524-32
19. Thirlaway K, Fallowfield L, Cuzick J. The Sexual Activity Questionnaire: a measure of women's sexual functioning. Qual Life Res 1996;**5**(1):81-90.
20. Freynhagen R, Baron R, Gockel U, et al. PainDETECT: a new screening questionnaire to identify neuropathic components in patients with back pain. Curr Med Res Opin 2006;**22**(10):1911-20.
21. Dworkin RH, Turk DC, Wyrwich KW, et al. Interpreting the clinical importance of treatment outcomes in chronic pain clinical trials: IMMPACT recommendations. J Pain 2008;**9**(2):105-21.
22. Grace VM. Problems women patients experience in the medical encounter for chronic pelvic pain: a New Zealand study. Health care for women international 1995;**16**(6):509-19.
23. Brawn J, Morotti M, Zondervan KT, et al. Central changes associated with chronic pelvic pain and endometriosis. Hum Reprod Update 2014;**20**(5):737-47.
24. Kaya S, Hermans L, Willems T, et al. Central sensitization in urogynecological chronic pelvic pain: a systematic literature review. Pain Physician 2013;**16**(4):291-308.
25. Whitaker LH, Reid J, Choa A, et al. An Exploratory Study into Objective and Reported Characteristics of Neuropathic Pain in Women with Chronic Pelvic Pain. PLoS One 2016;**11**(4):e0151950.
26. NICE. Neuropathic pain in adults: pharmacological management in non-specialist settings. NICE Clinical Guideline, 2013.
27. Dworkin RH, Turk DC, Farrar JT, et al. Core outcome measures for chronic pain clinical trials: IMMPACT recommendations. Pain 2005;**113**(1-2):9-19.
28. Dworkin RH, Turk DC, Peirce-Sandner S, et al. Research design considerations for confirmatory chronic pain clinical trials: IMMPACT recommendations. Pain 2010;**149**(2):177-93.

For peer review only

Authors' contributions

KV: research, contribution of original material, drafting, editing and approval of final manuscript; AH, SB, RC, JD, LM, WS: research, contribution of original material, editing and approval of final manuscript; AB, IT, JB, YC, BC, GM, AW: contribution of original material, editing and approval of final manuscript.

For peer review only - http://bmjopen.bmj.com/site/about/guidelines.xhtml

Funding statement

GaPP2 is funded by a grant from the NIHR/MRC Efficacy and Mechanism Evaluation (EME) Programme, Reference: 13/52/04.

Competing interests

KV has received research funding from Pfizer pharmaceuticals and Bayer Healthcare, honoraria from Bayer Healthcare, and consultancy fees from Grunenthal Gmb. AH receives grant funding from the NIHR and the Medical Research Council. APB has no competing issues as no direct funding from industry. APB is currently President of the British pain Society. JB declares no competing interests. SB receives grant funding from NIHR and Chief Scientist Office Scotland. He has been a speaker at a number of conferences that have received funding from the pharmaceutical industry. His clinical colleagues receive industry support for travel and for Departmental Seminars. AW has received a consulting fee from Astellas. GJM is Chief Investigator of the British Society for Rheumatology (BSR) Biologics Register in Ankylosing Spondylitis. This is funded by the BSR who receive funds from Pfizer, AbbVie and UCB. YC receives grant funding from NIHR. RC has secured research funding from Allergan and is a member of Allergan's scientific advisory panel. He also receives grant funding from the NIHR via UCL Biomedical Research Centre. IT is supported by grants from NIHR Oxford Biomedical Research Centre, Medical Research Council of Great Britain and Northern Ireland, the Wellcome Trust.

For peer review only

Table 1: Dose escalation schedule for GaPP2

Day in study	Total number of capsules/day (maximum)	Dosing	Maximum daily dose of gabapentin
1	1	1 capsule night	300 mg
2	1	1 capsule night	300 mg
3	1	1 capsule night	300 mg
4	2	1 capsule twice daily	600 mg
5	2	1 capsule twice daily	600 mg
6	2	1 capsule twice daily	600 mg
7	3	1 capsule three times daily	900 mg
8	3	1 capsule three times daily	900 mg
9	3	1 capsule three times daily	900 mg
10	4	1 capsule twice + 2 capsules at night	1200 mg
11	4	1 capsule twice + 2 capsules at night	1200 mg
12	4	1 capsule twice + 2 capsules at night	1200 mg
13	5	2 capsules twice + 1 capsule once	1500 mg
14	5	2 capsules twice + 1 capsule once	1500 mg
15	5	2 capsules twice + 1 capsule once	1500 mg
16	6	2 capsules three times daily	1800 mg
17	6	2 capsules three times daily	1800 mg
18	6	2 capsules three times daily	1800 mg
19	7	2 capsules twice + 3 capsules night	2100 mg
20	7	2 capsules twice + 3 capsules night	2100 mg
21	7	2 capsules twice + 3 capsules night	2100 mg
22	8	3 capsules twice + 2 capsules once	2400 mg
23	8	3 capsules twice + 2 capsules once	2400 mg
24	8	3 capsules twice + 2 capsules once	2400 mg
25	9	3 capsules three times daily	2700 mg
26	9	3 capsules three times daily	2700 mg
27	9	3 capsules three times daily	2700 mg
28 - 112	Remain on maximum tolerate dose until the end of week 16. (not exceeding 2700mg or 9 capsules per day). Daily dose should be divided equally into 3 doses.		

For peer review only

For peer review only - http://bmjopen.bmj.com/site/about/guidelines.xhtml

Table 2: Schedule of outcome assessments for GaPP2

Phase	Run-in	Baseline, randomisation & treatment dispensed	Titration	Treatment		End of study & unblinding	Taper
**Duration (weeks)**	-4 to -1	0	1-4	5-12	13-16		17-19
Weekly worst and average NRS	x x x x				x x x x		
SF12		X				X	
BPI		X				X	
PCQ		X				X	
SAQ		X				X	
BFI		X				X	
GHQ-12		X				X	
WPAIQ		X				X	
PainDETECT™		X				X	
PUF		X					
Adverse events			X	X	X	X	X
Permitted / Concomitant medication	X		X	X	X		X
Adherence or discontinuation			X	X	X		X

For peer review only

Figure legend

Figure 1: Study Flow chart.

For peer review only

For peer review only - http://bmjopen.bmj.com/site/about/guidelines.xhtml

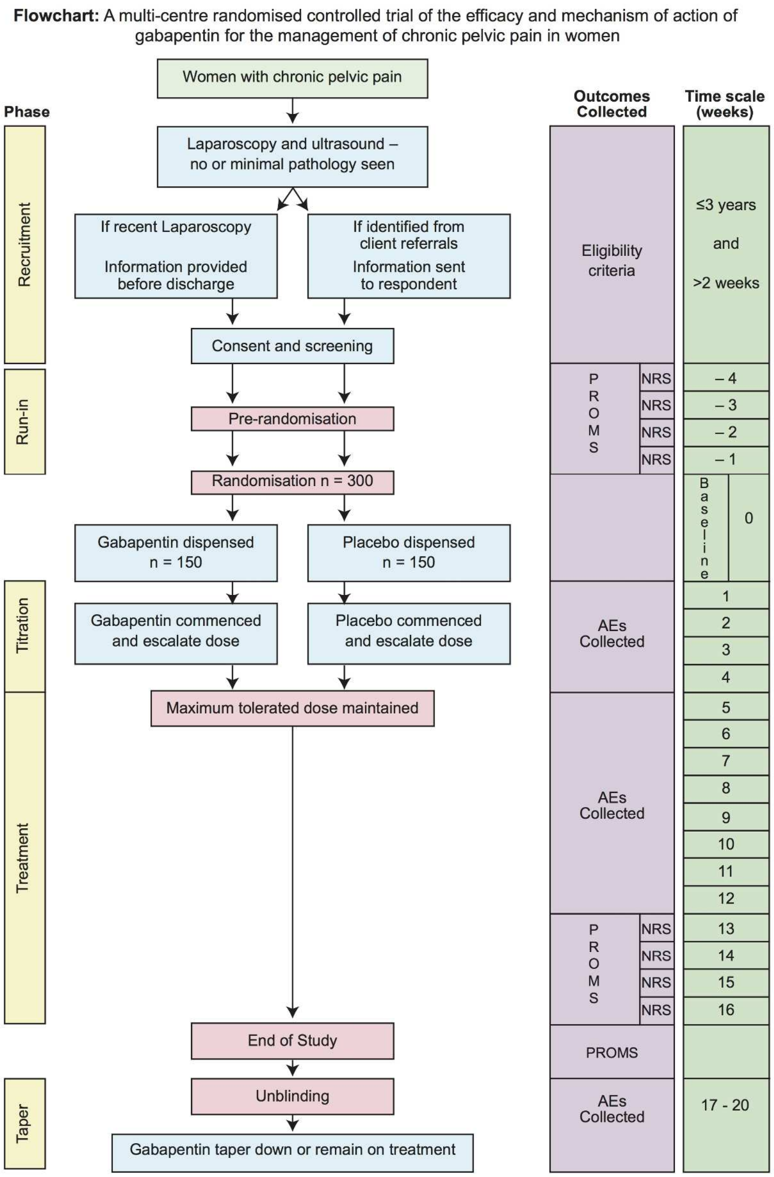

Figure 1

209x296mm (300 x 300 DPI)

SPIRIT

STANDARD PROTOCOL ITEMS: RECOMMENDATIONS FOR INTERVENTIONAL TRIALS

SPIRIT 2013 Checklist: Recommended items to address in a clinical trial protocol and related documents*

Section/item	ItemNo	Description	Addressed on page number
**Administrative information**			
Title	1	Descriptive title identifying the study design, population, interventions, and, if applicable, trial acronym	1
Trial registration	2a	Trial identifier and registry name. If not yet registered, name of intended registry	4
	2b	All items from the World Health Organization Trial Registration Data Set	4, 20, 17, 10, 8, 9, 7, 6
Protocol version	3	Date and version identifier	-
Funding	4	Sources and types of financial, material, and other support	20
Roles and responsibilities	5a	Names, affiliations, and roles of protocol contributors	1
	5b	Name and contact information for the trial sponsor	17
	5c	Role of study sponsor and funders, if any, in study design; collection, management, analysis, and interpretation of data; writing of the report; and the decision to submit the report for publication, including whether they will have ultimate authority over any of these activities	17
	5d	Composition, roles, and responsibilities of the coordinating centre, steering committee, endpoint adjudication committee, data management team, and other individuals or groups overseeing the trial, if applicable (see Item 21a for data monitoring committee)	17

For peer review only

For peer review only - http://bmjopen.bmj.com/site/about/guidelines.xhtml

**Introduction**			
Background and rationale	6a	Description of research question and justification for undertaking the trial, including summary of relevant studies (published and unpublished) examining benefits and harms for each intervention	5
	6b	Explanation for choice of comparators	11
Objectives	7	Specific objectives or hypotheses	6
Trial design	8	Description of trial design including type of trial (eg, parallel group, crossover, factorial, single group), allocation ratio, and framework (eg, superiority, equivalence, noninferiority, exploratory)	7-8
**Methods: Participants, interventions, and outcomes**			
Study setting	9	Description of study settings (eg, community clinic, academic hospital) and list of countries where data will be collected. Reference to where list of study sites can be obtained	10
Eligibility criteria	10	Inclusion and exclusion criteria for participants. If applicable, eligibility criteria for study centres and individuals who will perform the interventions (eg, surgeons, psychotherapists)	8-9
Interventions	11a	Interventions for each group with sufficient detail to allow replication, including how and when they will be administered	10
	11b	Criteria for discontinuing or modifying allocated interventions for a given trial participant (eg, drug dose change in response to harms, participant request, or improving/worsening disease)	14
	11c	Strategies to improve adherence to intervention protocols, and any procedures for monitoring adherence (eg, drug tablet return, laboratory tests)	12
	11d	Relevant concomitant care and interventions that are permitted or prohibited during the trial	10

For peer review only - http://bmjopen.bmj.com/site/about/guidelines.xhtml

Outcomes	12	Primary, secondary, and other outcomes, including the specific measurement variable (eg, systolic blood pressure), analysis metric (eg, change from baseline, final value, time to event), method of aggregation (eg, median, proportion), and time point for each outcome. Explanation of the clinical relevance of chosen efficacy and harm outcomes is strongly recommended	6-7
Participant timeline	13	Time schedule of enrolment, interventions (including any run-ins and washouts), assessments, and visits for participants. A schematic diagram is highly recommended (see Figure)	23, 25
Sample size	14	Estimated number of participants needed to achieve study objectives and how it was determined, including clinical and statistical assumptions supporting any sample size calculations	15
Recruitment	15	Strategies for achieving adequate participant enrolment to reach target sample size	9
**Methods: Assignment of interventions (for controlled trials)**			
Allocation:			
Sequence generation	16a	Method of generating the allocation sequence (eg, computer-generated random numbers), and list of any factors for stratification. To reduce predictability of a random sequence, details of any planned restriction (eg, blocking) should be provided in a separate document that is unavailable to those who enrol participants or assign interventions	10
Allocation concealment mechanism	16b	Mechanism of implementing the allocation sequence (eg, central telephone; sequentially numbered, opaque, sealed envelopes), describing any steps to conceal the sequence until interventions are assigned	10
Implementation	16c	Who will generate the allocation sequence, who will enrol participants, and who will assign participants to interventions	10
Blinding (masking)	17a	Who will be blinded after assignment to interventions (eg, trial participants, care providers, outcome assessors, data analysts), and how	10

For peer review only - http://bmjopen.bmj.com/site/about/guidelines.xhtml

	17b	If blinded, circumstances under which unblinding is permissible, and procedure for revealing a participant's allocated intervention during the trial	14
**Methods: Data collection, management, and analysis**			
Data collection methods	18a	Plans for assessment and collection of outcome, baseline, and other trial data, including any related processes to promote data quality (eg, duplicate measurements, training of assessors) and a description of study instruments (eg, questionnaires, laboratory tests) along with their reliability and validity, if known. Reference to where data collection forms can be found, if not in the protocol	11-13, 25
	18b	Plans to promote participant retention and complete follow-up, including list of any outcome data to be collected for participants who discontinue or deviate from intervention protocols	14, 16
Data management	19	Plans for data entry, coding, security, and storage, including any related processes to promote data quality (eg, double data entry; range checks for data values). Reference to where details of data management procedures can be found, if not in the protocol	11
Statistical methods	20a	Statistical methods for analysing primary and secondary outcomes. Reference to where other details of the statistical analysis plan can be found, if not in the protocol	15-17
	20b	Methods for any additional analyses (eg, subgroup and adjusted analyses)	16
	20c	Definition of analysis population relating to protocol non-adherence (eg, as randomised analysis), and any statistical methods to handle missing data (eg, multiple imputation)	16
**Methods: Monitoring**			
Data monitoring	21a	Composition of data monitoring committee (DMC); summary of its role and reporting structure; statement of whether it is independent from the sponsor and competing interests; and reference to where further details about its charter can be found, if not in the protocol. Alternatively, an explanation of why a DMC is not needed	17

	21b	Description of any interim analyses and stopping guidelines, including who will have access to these interim results and make the final decision to terminate the trial	-
Harms	22	Plans for collecting, assessing, reporting, and managing solicited and spontaneously reported adverse events and other unintended effects of trial interventions or trial conduct	14
Auditing	23	Frequency and procedures for auditing trial conduct, if any, and whether the process will be independent from investigators and the sponsor	17
**Ethics and dissemination**			
Research ethics approval	24	Plans for seeking research ethics committee/institutional review board (REC/IRB) approval	17
Protocol amendments	25	Plans for communicating important protocol modifications (eg, changes to eligibility criteria, outcomes, analyses) to relevant parties (eg, investigators, REC/IRBs, trial participants, trial registries, journals, regulators)	17
Consent or assent	26a	Who will obtain informed consent or assent from potential trial participants or authorised surrogates, and how (see Item 32)	9
	26b	Additional consent provisions for collection and use of participant data and biological specimens in ancillary studies, if applicable	-
Confidentiality	27	How personal information about potential and enrolled participants will be collected, shared, and maintained in order to protect confidentiality before, during, and after the trial	11
Declaration of interests	28	Financial and other competing interests for principal investigators for the overall trial and each study site	21
Access to data	29	Statement of who will have access to the final trial dataset, and disclosure of contractual agreements that limit such access for investigators	17
Ancillary and post-trial care	30	Provisions, if any, for ancillary and post-trial care, and for compensation to those who suffer harm from trial participation	14

For peer review only - http://bmjopen.bmj.com/site/about/guidelines.xhtml

Dissemination policy	31a	Plans for investigators and sponsor to communicate trial results to participants, healthcare professionals, the public, and other relevant groups (eg, via publication, reporting in results databases, or other data sharing arrangements), including any publication restrictions	17
	31b	Authorship eligibility guidelines and any intended use of professional writers	17
	31c	Plans, if any, for granting public access to the full protocol, participant-level dataset, and statistical code	-
**Appendices**			
Informed consent materials	32	Model consent form and other related documentation given to participants and authorised surrogates	-
Biological specimens	33	Plans for collection, laboratory evaluation, and storage of biological specimens for genetic or molecular analysis in the current trial and for future use in ancillary studies, if applicable	-

*It is strongly recommended that this checklist be read in conjunction with the SPIRIT 2013 Explanation & Elaboration for important clarification on the items. Amendments to the protocol should be tracked and dated. The SPIRIT checklist is copyrighted by the SPIRIT Group under the Creative Commons "Attribution-NonCommercial-NoDerivs 3.0 Unported" license.

For peer review only

For peer review only - http://bmjopen.bmj.com/site/about/guidelines.xhtml

BMJ Open

GaPP2: A multi-centre randomised controlled trial of the efficacy of gabapentin for the management of chronic pelvic pain in women: study protocol

Journal:	*BMJ Open*
Manuscript ID	bmjopen-2016-014924.R2
Article Type:	Protocol
Date Submitted by the Author:	23-Sep-2017
Complete List of Authors:	Vincent, Katy; University of Oxford, Nuffield Department of Obstetrics and Gyanecology<br>Baranowski, Andrew ; The National Hospital for Neurology and Neurosurgery , Pain Management Centre<br>Bhattacharya, Siladitya; Aberdeen University, Obstetrics and Gynaecology<br>Birch, Judy; Pelvic Pain Support Network<br>Cheong, Ying; University of Southampton Faculty of Medicine<br>Cregg, Roman; The National Hospital for Neurology and Neurosurgery, Pain Management Centre<br>Daniels, Jane; University of Birmingham, Birmingham Clinical Trials Unit<br>Hewitt, Catherine; University of Birmingham, Clinical Trials Unit<br>Macfarlane, Gary; University of Aberdeen, Epidemiology Group<br>Middleton, Lee; University of Birmingham, Clinical Trials Unit<br>Szubert, Wojciech; University of Edinburgh, MRC Centre for Reproductive Health<br>Tracey, Irene; University of Oxford, Nuffield Department of Clinical Neurosciences<br>Williams, Amanda; University College London, Research Department of Clinical, Educational and Health Psychology<br>Horne, Andrew
**Primary Subject Heading**:	Obstetrics and gynaecology
Secondary Subject Heading:	Evidence based practice, Anaesthesia
Keywords:	gabapentin, chronic pelvic pain, Pain management < ANAESTHETICS

SCHOLARONE™ Manuscripts

TITLE PAGE

Title: GaPP2: A multi-centre randomised controlled trial of the efficacy of gabapentin for the management of chronic pelvic pain in women: study protocol

Authors: Katy Vincent[1], Andrew Baranowski[2], Siladitya Bhattacharya[3], Judy Birch[4], Ying Cheong[5], Roman Cregg[2], Jane Daniels[6], Catherine A. Hewitt[6], Gary J Macfarlane[7], Lee Middleton[6], Wojciech Szubert[8], Irene Tracey[9], Amanda C de C Williams[10], Andrew W Horne[8]

[1] Nuffield Department of Obstetrics and Gynaecology, University of Oxford, OX3 9DU, UK.

[2] Pain Management Centre, The National Hospital for Neurology and Neurosurgery, London W1T 4AJ, UK.

[3] Institute of Applied Health Sciences, School of Medicine, Medical Sciences and Nutrition, University of Aberdeen, UK

[4] Pelvic Pain Support Network, Poole, BH17 9AT, UK.

[5] Faculty of Medicine, University of Southampton, SO16 5YA, UK.

[6] Birmingham Clinical Trials Unit, University of Birmingham. B15 2TT. UK

[7] Epidemiology Group, School of Medicine, Medical Sciences and Nutrition, University of Aberdeen, UK

[8] MRC Centre for Reproductive Health, Queen's Medical Research Institute, 47 Little France Crescent, Edinburgh EH16 4TJ

[9] Nuffield Department of Clinical Neurosciences, University of Oxford, OX3 9DU, UK.

[10] Research Dept of Clinical, Educational & Health Psychology, University College London, Gower St, London WC1E 6BT

For peer review only - http://bmjopen.bmj.com/site/about/guidelines.xhtml

Corresponding author:

Professor Andrew Horne

MRC Centre for Reproductive Health

Queen's Medical Research Institute

47 Little France Crescent

Edinburgh EH16 4TJ

Tel: +44 131 242 6988

Email: andrew.horne@ed.ac.uk

Key Words: Chronic Pelvic Pain; Gabapentin; Randomised Controlled Trial

Word Count: 3380

For peer review only

ABSTRACT

Introduction:

Chronic pelvic pain (CPP) affects more than 1 million UK women with associated healthcare costs of £158 million annually. Current evidence supporting interventions when no underlying pathology is identified is very limited and treatment is frequently inadequate. Gabapentin (a GABA analogue) is efficacious and often well tolerated in other chronic pain conditions. We have completed a successful pilot randomised controlled trial (GaPP1) and here describe the protocol for the definitive multicentre trial to assess the efficacy of gabapentin in the management of CPP in women (GaPP2).

Methods and analysis:

We plan to perform a double blind placebo controlled randomised multi-centre clinical trial, recruiting 300 women with CPP from more than 8 NHS hospitals within the UK. After randomisation, women will titrate their medication (gabapentin or placebo) over a 4-week period to a maximum of 2700mg or placebo equivalent and will then maintain a stable dose for a 12 week period. Response to treatment will be monitored with validated questionnaires and co-primary outcome measures of average and worst pain scores will be employed. The primary objective is to test the hypothesis that treatment with gabapentin has the potential to provide an effective oral treatment to alleviate pain in women with CPP in the absence of any obvious pelvic pathology.

Ethics and dissemination:

Ethical approval has been obtained from the Coventry and Warwick Research Ethics Committee (REC 15/WM/0036). Data will be presented at international conferences

For peer review only - http://bmjopen.bmj.com/site/about/guidelines.xhtml

and published in peer-reviewed journals. We will make the information obtained from the study available to the public through national bodies and charities.

Trial registration number: ISRCTN77451762

For peer review only

For peer review only - http://bmjopen.bmj.com/site/about/guidelines.xhtml

INTRODUCTION

Chronic pelvic pain (CPP) affects more than 1 million women in the UK[1-3]. It is associated with significantly reduced quality of life (QoL)[4 5], a 45% reduction in work productivity and it has been estimated that caring for women with CPP in the UK costs £158 million annually[6 7]. CPP can be associated with underlying pathology such as endometriosis, but in up to 55% of women no obvious cause can be identified at laparoscopy[6]. Management of CPP is difficult when no pathology is identified, as no established gynaecological treatments are available. Due to its effectiveness in other chronic pain conditions, gabapentin (a GABA analogue), is increasingly being prescribed for CPP in both primary and secondary care[8]. However, there is no good quality evidence in CPP specifically on which to base this practice[9]. To our knowledge, there is only one study evaluating the use of gabapentin for CPP, which did not have a placebo arm[10]. This small study in 56 women, compared gabapentin to amitriptyline and showed gabapentin to have greater efficacy at improving pain scores at 12 months. However, efficacy of gabapentin has been proven in other chronic pain conditions. A recent high quality review showed the number needed to treat (NNT) to be 5.8 (95% CI 4.3 to 9.0) to achieve at least 50% pain intensity reduction in painful diabetic neuropathy (829 patients); 7.5 (95% CI 5.2 to 14) to achieve at least 50% pain intensity reduction in postherpetic neuralgia (892 patients); and 5.4 (95% CI 2.9 to 31) to achieve at least 30% pain intensity reduction in fibromyalgia (150 patients)[8]. Moreover it is a drug that is very well tolerated: all-cause withdrawal rates are similar to placebo (gabapentin: 20%; placebo: 19%; number of studies: 17; number of participants: 3063)[8].

For peer review only - http://bmjopen.bmj.com/site/about/guidelines.xhtml

Given the clinical need for a medical treatment for CPP with no identifiable underlying pathology and the strong evidence supporting the acceptability and efficacy of gabapentin in other chronic pain conditions, we considered further investigation of gabapentin as a potential treatment for CPP in women was warranted. We hypothesise that treatment of women with CPP in the absence of any obvious pelvic pathology with gabapentin will alleviate pain and improve physical and emotional functioning. We initially performed a successful pilot randomised controlled trial (RCT) (GaPP1)[11 12]. Here we describe the protocol for our definitive multicentre trial to assess the efficacy of gabapentin in the management of CPP in women (GaPP2).

Objectives

Primary objective

The primary objective is to test the hypothesis that treatment with gabapentin has the potential to provide an effective oral treatment to alleviate pain in women with CPP in the absence of any obvious pelvic pathology.

Secondary objective

The secondary objective is to test the hypothesis that treatment with gabapentin has the potential to improve physical and emotional functioning in women with CPP in the absence of any obvious pelvic pathology.

Outcomes

Primary outcome

We will employ co-primary outcome measures of average and worst pain scores recorded on a numerical rating scale (NRS). To capture the cyclicity that may occur

For peer review only - http://bmjopen.bmj.com/site/about/guidelines.xhtml
For peer review only

with CPP, weekly pain scores (on a 0-10 scale) will be recorded during the final four weeks of treatment (weeks 13-16 post randomisation), in the form of:

i) ‘average pain this week’ and

ii) ‘worst pain this week’

The composite ‘average’ pain score will be taken as the average of the four weekly average pain scores submitted, and the composite ‘worst’ pain score as the worst of the four weekly worst pain scores submitted.

For peer review only

Secondary outcomes

- Physical and emotional function and quality of life
- Satisfaction with treatment
- Patient estimate of whether on active treatment or on placebo group, and confidence in and reasons for estimate
- Adherence to trial treatments, as reported by the participants
- Concomitant analgesic use, as reported by the participants
- Adverse events, as reported by participants (principally those that are serious and detailed in the summary of product characteristics and those that are unexpected)
- GP/hospital consultations, as reported by the participants
- Time off work and ‘presenteeism’

METHODS AND ANALYSIS

Study Design

GaPP2 is a double blind placebo controlled randomised multi-centre clinical trial (Figure 1). We will screen women with CPP from more than 8 NHS hospitals within

For peer review only - http://bmjopen.bmj.com/site/about/guidelines.xhtml

the United Kingdom. Women will return weekly NRS pain scores to the trials office for 4 weeks after initial consent. Those women meeting the inclusion criteria at the end of these 4 weeks will be randomised. We will randomise 300 women (150 to gabapentin, 150 to placebo). After randomisation and titration, participants will receive treatment with the maximum tolerated dose for 12 weeks. Participants and the healthcare team will be unblinded at the end of their treatment.

Participants

A total of 300 women with a history of chronic pelvic pain with no obvious pelvic pathology detected at laparoscopy will be recruited to the trial.

Inclusion criteria

- Women aged between 18-50 years
- CPP (~~non-cyclical~~ with or without dysmenorrhoea or dyspareunia) of >3 months duration
- Pain located within the true pelvis or between and below anterior iliac crests
- No obvious pelvic pathology at laparoscopy (laparoscopy must have taken place at least 2 weeks ago, but no more than 36 months prior to screening)
- Using or willing to use effective contraception if necessary to avoid pregnancy
- Able to give informed consent
- For both the worst and average pre-randomisation NRS questions, at least three of the four weekly scores returned to the trials office. At least two of the worst pain scores should be ≥4/10. Potential participants who have been on a stable dose of an analgesic, other than gabapentin or pregabalin, for at least 4 weeks prior to screening will be eligible.

For peer review only - http://bmjopen.bmj.com/site/about/guidelines.xhtml

For peer review only

Exclusion criteria

- Dysmenorrhoea alone
- Known pelvic pathology:
  - Endometriosis (macroscopic lesions)
  - complex or >5cm ovarian cyst
  - fibroid >3cm
  - dense adhesions
- Current malignancy under treatment
- Current use of gabapentin/pregabalin.
- Taking GnRH agonists and unable/unwilling to stop
- Surgery planned in the next 6 months
- History of significant renal impairment
- Previous allergic reaction to gabapentin
- Breast feeding
- Pregnant
- Planning pregnancy in next 6 months
- Pain suspected to be of gastrointestinal origin (positive Rome III Diagnostic Criteria)
- Co-enrolment in another Clinical Trial of an Investigational Medicinal Product

Participant enrolment

Research nurses (dedicated or through the National Institute for Health Research's Clinical Research Network, depending on the site) will be employed for the duration of the study to approach eligible women, provide them with patient information sheets and offer them the opportunity to discuss the trial, and obtain informed consent for

For peer review only - http://bmjopen.bmj.com/site/about/guidelines.xhtml

screening. Consent will only be taken once the patient has had ample time to read the patient information sheet and had her questions answered.

Study settings

We will recruit patients from gynaecology outpatient clinics, gynaecology wards and day surgery units and chronic pelvic pain clinics within the UK.

Intervention and randomisation

Randomisation to gabapentin or placebo will occur once written informed consent has been obtained, final eligibility established from the pain responses provided during the screening phase, and baseline questionnaires completed. The Birmingham Clinical Trials Unit (BCTU) will provide third party web-based randomisation with telephone back-up. A minimisation procedure using a computer based algorithm will be used to avoid chance imbalances in treatment allocation and the following potentially important variables:

1. Presence or absence of dysmenorrhoea (a pain score of ≥4/10 will be considered significant)
2. Psychological distress measured by the General Health Questionnaire (scored as 0-12 with a cut off of 0-1 and 2-12 for minimisation)
3. Use of sex hormonal treatments (combined oral contraceptive, progestogens, levonorgestrel-releasing intrauterine system (Mirena®)).
4. Centre

A 'random element' will be included in the minimisation algorithm, so that each patient has a probability (unspecified here), of being randomised to the opposite treatment that they would have otherwise received. Full details of the algorithm used

For peer review only - http://bmjopen.bmj.com/site/about/guidelines.xhtml

For peer review only

will be stored in a confidential document at BCTU. Both participants and the research team will remain blind to allocation.

Dose regimen

After randomisation, participants will be allocated a trial treatment pack from the hospital pharmacy containing either gabapentin or placebo oral tablets, both of identical appearance. Participants will start on 1 capsule (300mgs) daily and will increase by 1 capsule (300 mgs) increments every three days until they perceive that they are gaining adequate pain relief, or report side effects (e.g. dizziness, somnolence, mood changes, appetite and poor concentration) that preclude them from further increases, up to a maximum dose of 9 capsules (2700 mgs), as shown in Table 1. The titration phase will last a maximum of 4 weeks. If necessary they will be advised to titrate down to the last tolerated dose with minimal side effects. They will be asked to maintain their best tolerated dose until the end of week 16. Patients will be advised and given written instructions regarding their dosing regimen by a member of the research team. It will be recommended that the drug should be taken in three equally divided doses daily. The same protocol will be used for the placebo. When the participant stops treatment the dose will be reduced according to a dose reduction chart and written instructions will be given. Patients will be allowed to use other medication (including analgesics, self-medication and alternative treatments, e.g. acupuncture) throughout the study period.

For peer review only

For peer review only - http://bmjopen.bmj.com/site/about/guidelines.xhtml

Data collection

Data storage

All the data generated from the study will be stored in a bespoke database, which will be password protected. All paperwork will be kept in a locked filing cabinet in a locked office. All data will be stored in accordance with the Data Protection Act.

Screening

A member of the research team will assess the woman for eligibility to enter the screening phase. All data will be recorded on a CRF and transferred to a secure database, which will trigger the start of the weekly collection of pain scores.

Participant log

The clinical research team will keep an anonymised electronic log of women who fulfil the eligibility criteria, women who are invited to participate in the study, women recruited and women who leave the trial early. Reasons for non-recruitment (e.g. non-eligibility, refusal to participate, administrative error) will also be recorded. During the course of the study, we will document reasons for withdrawal from the study and loss to follow-up if available.

Pain scores

Pain NRS will be collected by an automated text messaging system. Two texts will be sent to the women's mobile phone, asking about average and worst pain respectively, and the woman will be asked to reply to the text message with her pain score, rating it from zero for no pain at all, to 10 being worst pain imaginable. To capture cyclicity, these will be collected weekly during the eligibility phase (weeks -1 – -4) and during the last 4 weeks of the treatment phase (weeks 13 – 16).

For peer review only

For peer review only - http://bmjopen.bmj.com/site/about/guidelines.xhtml

Treatment Diaries

Participants will be provided with a treatment diary at the same time as their medication pack is dispensed. The following measures will be completed by the participant daily from day 1 of treatment until week 16:

- Dose of gabapentin taken
- Reason for any change in trial medication dose
- Alternative therapies used
- Any visits to a healthcare professional

Questionnaires

A questionnaire will be given to all participants before randomisation but after screening (Baseline) and at 16 weeks post randomisation (See Table 2 for full schedule of assessments). This will include the following validated tools:

- 12-Item Short-Form Health Survey (SF-12): a quality of life measure[13].
- Brief Pain Inventory (BPI)[14]: a tool to measure pain intensity and interference of pain in a patient's life.
- Brief Fatigue Inventory (BFI)[15].
- General Health Questionnaire (GHQ)[16]: to identify psychological distress.
- Work and Productivity Activity Impairment (WPAI)[17].
- Pain catastrophising scale (PCS)[18].
- Sexual Activity Questionnaire (SAQ)[19].
- PainDETECT™: to identify a neuropathic component to pain[20].
- Pelvic Pain and Urinary/ Frequency Patient Symptom Scale (PUF) (at baseline only)

The questionnaire at baseline will include questions to capture the baseline demographic and clinical characteristics of the participants.

For peer review only - http://bmjopen.bmj.com/site/about/guidelines.xhtml
For peer review only

All questionnaires will be anonymised and completed in private.

Adverse Events

Participants will collect information about adverse events in their treatment diaries. However, they will be instructed to contact the clinical research team at any time after consenting to join the trial if they have an event that requires hospitalisation or an event that results in persistent or significant disability or incapacity. Any serious adverse events that occur after joining the trial will be reported in detail in the participant's medical notes and followed up until resolution of the event. The assessment of seriousness, causality and expectedness will be conducted assuming that the participant received gabapentin, with the blinding not broken. All SAEs will be reported to the ACCORD Research Governance (http://www.accord.ed.ac.uk) and QA Office based at the University of Edinburgh immediately or within 24 hours. ACCORD will onward report all SAEs to BCTU within 7 days.

Termination of Study

Participants will be unblinded at the end of the study and if taking gabapentin will have the option to continue on treatment or will be tapered off treatment. Participants who have been on placebo will be given the choice to start on gabapentin, which will be prescribed by their clinician. Participants will be given an emergency contact card to carry while participating in the study. The blinding code will only be broken in emergency situations for reasons of patient safety, where knowledge of the treatment administered is necessary for the treatment of a serious adverse event. Participants whose randomisation codes are broken will cease treatment with the study drug, but will continue to be followed up. Participants may discontinue from the trial at any time at their own request, or they may be withdrawn at any time at the discretion of

For peer review only

For peer review only - http://bmjopen.bmj.com/site/about/guidelines.xhtml

the research team for safety, behavioural or administrative reasons. Data collection is envisaged to be complete in September 2018.

Sample size

We have based our sample size on being able to detect a minimally important difference (MID) in NRS scores with high levels of power. Studies have shown the MID in this population to be around 1 point on a 0-10 scale [21]. Our pilot study showed worst and average pain scores to have standard deviations between 2.0 and 2.5. If the SD is at the lower end of these estimates, 86 patients in each group (172 in total) would be required to have 90% power (p=0.05) to detect a difference of 1 point. If the SD is at the higher end, we could detect the same difference with 80% power (p=0.05) with 100 patients in each group. We have assumed the latter SD (2.5) to be conservative. To account for any increase in the risk of type I error that may be associated with having co-primary outcome measures we have applied a Bonferroni correction (alpha reduced to 0.025 from 0.05), which increases the sample size to 120 per group. Furthermore, to account for an expected average 20% loss to follow-up we will randomise 150 per group, 300 patients in total.

Proposed Analyses

Data analysis will be by intention to treat. Every attempt will be made to gather data on all women randomised, irrespective of compliance with the treatment protocol. Appropriate baseline characteristics, split by treatment group, will be presented for each outcome. Point estimates, 95% confidence intervals and p-values from two-sided tests will be reported.

For peer review only - http://bmjopen.bmj.com/site/about/guidelines.xhtml

Primary analysis

We will use a linear regression model to estimate differences in worst and average NRS scores between the two treatment groups, including baseline score and the minimisation variables as covariates. The p-value from the associated chi-squared test will be produced and used to determine statistical significance. A Bonferroni correction will be applied as there are two primary outcomes. Further analysis using a repeated measures (multi-level) model will also be performed incorporating all eight recorded scores.

Secondary analysis

Data from the other continuous measures (SF-12, BPI, PCQ, SAQ, WPAIQ, BFI, PainDETECT™ and GHQ) will be analysed in a similar manner to the primary measure. Other outcome measures (use of permitted analgesic medication, satisfaction) will be analysed using standard methods (tests for trend, absolute/relative risks). Further analysis on pain scores will include an examination of the proportion of women that have a 30% and a 50% reduction in average and worst score from baseline as the outcome. A log-binomial regression model will be used here to generate adjusted relative risks. Sub-group analyses will be limited to the same variables that were used as minimisation variables. Tests for statistical heterogeneity (e.g. by including treatment group by subgroup interaction parameter in the linear regression model) will be performed prior to any examination of effect estimate within subgroups. In addition, we will investigate up to nine clinical variables measured at baseline to determine whether they correlate with response to treatment. These will include the minimisation variables (presence of dysmenorrhoea/psychological distress/current use of hormonal treatment) along with measures of intensity and of nature of pain (e.g. PainDETECT™), number of

For peer review only - http://bmjopen.bmj.com/site/about/guidelines.xhtml

functional systems involved (as a measure of organ specific versus generalised pelvic pain syndrome) and PUF score.

Missing data and sensitivity analyses

Every attempt will be used to collect full follow up data on all women. In particular, participants will continue to be followed up even after protocol treatment violation. It is thus anticipated that missing data will be minimal. Patients with completely missing primary outcome data or with only one of four pain scores recorded will not be included in the primary analysis. Secondary sensitivity analyses will be performed to investigate the impact of missing data for the primary outcome: this will include a worst score assumption. We will also simulate missing responses using a multiple imputation approach.

ETHICS AND DISSEMINATION

Ethical approval has been obtained from the Coventry and Warwick Research Ethics Committee (REC 15/WM/0036). Data will be presented at international conferences and published in peer-reviewed journals. We will make the information obtained from the study available to the public through relevant national bodies and charities.

RESEARCH GOVERNANCE

We shall adopt the standard approach used for monitoring randomised controlled trials and have a Trial Steering Committee (TSC) of at least four independent members, including pain specialist, a gynaecologist, trial methodologist and a PPI representative. There will also be a Data Monitoring Committee (DMC) comprising three independent members (a pain specialist, a gynaecologist and a statistician with extensive trial experience) who will review interim analyses. The terms of reference

For peer review only - http://bmjopen.bmj.com/site/about/guidelines.xhtml

For peer review only

and charter for this DMC will be guided by the DAMOCLES project, and we anticipate the DMC and TSC will meet biannually.

DISCUSSION

CPP is a major public health issue for women throughout the developed world[2]. As with other chronic pain conditions it is associated with a marked reduction in quality of life and significant financial costs for the woman, her family and society as a whole[4 5]. When CPP is associated with underlying pathology such as endometriosis, therapies targeting the pathology can be initiated. However, in more than 50% of women no underlying cause is identified[6]. For these women, not only is it difficult to comprehend and come to terms with how there can be no associated pathology[22], there are also no available evidence-based treatments to consider.

The efficacy of a number of pharmacological and interventional therapies has been investigated for other chronic pain syndromes. There is increasing evidence that women with CPP demonstrate central changes similar to those associated with other forms of chronic pain[23 24] and thus it is likely that such therapies would also be effective for CPP. Moreover, recent work demonstrates a neuropathic component in a significant proportion of women with CPP[25], further supporting the investigation of drugs currently recommended for neuropathic pain[26] in women with CPP. The multicentre placebo-controlled RCT described here aims to contribute to the evidence base by assessing the efficacy of gabapentin in women with CPP with no underlying pathology[9]. This trial is designed in line with the IMMPACT recommendations for the design of trials in chronic pain conditions[21 27 28] and builds upon a successful pilot study[11 12]. Women with CPP were surveyed to identify whether reduction in average

For peer review only - http://bmjopen.bmj.com/site/about/guidelines.xhtml

or worst pain was most important to them. As there was no clear consensus (average 43.4%, worst 56.6%) co-primary outcomes of average and worst pain scores have been chosen. We envisage the findings being of relevance to both primary and secondary care clinicians managing women with CPP.

REFERENCES

1. Zondervan KT, Yudkin PL, Vessey MP, et al. Prevalence and incidence of chronic pelvic pain in primary care: evidence from a national general practice database. British Journal of Obstetrics and Gynaecology 1999;**106**(11):1149-55.
2. Latthe P, Latthe M, Say L, et al. WHO systematic review of prevalence of chronic pelvic pain: a neglected reproductive health morbidity. BMC Public Health 2006;**6**:177.
3. Donaldson L. 150 years of the Annual Report of the Chief Medical Officer: On the state of public health 2008. London: DoH, 2009.
4. Breivik H, Collett B, Ventafridda V, et al. Survey of chronic pain in Europe: prevalence, impact on daily life, and treatment. Eur J Pain 2006;**10**(4):287-333.
5. Ayorinde AA, Macfarlane GJ, Saraswat L, et al. Chronic pelvic pain in women: an epidemiological perspective. Womens Health (Lond Engl) 2015;**11**(6):851-64.
6. Daniels JP, Khan KS. Chronic pelvic pain in women. BMJ 2010;**341**:c4834.
7. Cheong Y, William Stones R. Chronic pelvic pain: aetiology and therapy. Best Pract Res Clin Obstet Gynaecol 2006;**20**(5):695-711.
8. Wiffen PJ, Derry S, Moore RA, et al. Antiepileptic drugs for neuropathic pain and fibromyalgia - an overview of Cochrane reviews. Cochrane Database Syst Rev 2013;**11**:CD010567.
9. Horne AW, Vincent K, Cregg, Daniels J. Is gabapentin effective for women with unexplained chronic pelvic pain? BMJ (in press - publication date 23 09 17)
10. Sator-Katzenschlager SM, Scharbert G, Kress HG, et al. Chronic pelvic pain treated with gabapentin and amitriptyline: a randomized controlled pilot study. Wiener klinische Wochenschrift 2005;**117**(21-22):761-8.
11. Horne AW, Critchley HO, Doust A, et al. GaPP: a pilot randomised controlled trial of the efficacy of action of gabapentin for the management of chronic pelvic pain in women: study protocol. BMJ Open 2012;**2**(3).
12. Lewis SC, Bhattacharya S, Wu O, et al. Gabapentin for the Management of Chronic Pelvic Pain in Women (GaPP1): A Pilot Randomised Controlled Trial. PLoS One 2016;**11**(4):e0153037.
13. Ware J, Jr., Kosinski M, Keller SD. A 12-Item Short-Form Health Survey: construction of scales and preliminary tests of reliability and validity. Med Care 1996;**34**(3):220-33.
14. Cleeland CS, Ryan KM. Pain assessment: global use of the Brief Pain Inventory. Annals of the Academy of Medicine, Singapore 1994;**23**(2):129-38.

For peer review only - http://bmjopen.bmj.com/site/about/guidelines.xhtml

15. Mendoza TR, Wang XS, Cleeland CS, et al. The rapid assessment of fatigue severity in cancer patients: use of the Brief Fatigue Inventory. Cancer 1999;**85**(5):1186-96.
16. Goldberg DP. *Manual of the General Health Questionnaire*. Windsor, England: NFER Publishing, 1978.
17. Reilly MC, Zbrozek AS, Dukes EM. The validity and reproducibility of a work productivity and activity impairment instrument. Pharmacoeconomics 1993;**4**(5):353-65.
18. Sullivan MJL, Bishop SR, Pivik J. The Pain Catastrophizing Scale: Development and validation. Psychol Assess 1995;**7**(4):524-32
19. Thirlaway K, Fallowfield L, Cuzick J. The Sexual Activity Questionnaire: a measure of women's sexual functioning. Qual Life Res 1996;**5**(1):81-90.
20. Freynhagen R, Baron R, Gockel U, et al. PainDETECT: a new screening questionnaire to identify neuropathic components in patients with back pain. Curr Med Res Opin 2006;**22**(10):1911-20.
21. Dworkin RH, Turk DC, Wyrwich KW, et al. Interpreting the clinical importance of treatment outcomes in chronic pain clinical trials: IMMPACT recommendations. J Pain 2008;**9**(2):105-21.
22. Grace VM. Problems women patients experience in the medical encounter for chronic pelvic pain: a New Zealand study. Health care for women international 1995;**16**(6):509-19.
23. Brawn J, Morotti M, Zondervan KT, et al. Central changes associated with chronic pelvic pain and endometriosis. Hum Reprod Update 2014;**20**(5):737-47.
24. Kaya S, Hermans L, Willems T, et al. Central sensitization in urogynecological chronic pelvic pain: a systematic literature review. Pain Physician 2013;**16**(4):291-308.
25. Whitaker LH, Reid J, Choa A, et al. An Exploratory Study into Objective and Reported Characteristics of Neuropathic Pain in Women with Chronic Pelvic Pain. PLoS One 2016;**11**(4):e0151950.
26. NICE. Neuropathic pain in adults: pharmacological management in non-specialist settings. NICE Clinical Guideline, 2013.
27. Dworkin RH, Turk DC, Farrar JT, et al. Core outcome measures for chronic pain clinical trials: IMMPACT recommendations. Pain 2005;**113**(1-2):9-19.
28. Dworkin RH, Turk DC, Peirce-Sandner S, et al. Research design considerations for confirmatory chronic pain clinical trials: IMMPACT recommendations. Pain 2010;**149**(2):177-93.

For peer review only

Authors' contributions

KV: research, contribution of original material, drafting, editing and approval of final manuscript; AH, SB, RC, JD, LM, WS: research, contribution of original material, editing and approval of final manuscript; AB, IT, JB, YC, BC, GM, AW: contribution of original material, editing and approval of final manuscript.

For peer review only - http://bmjopen.bmj.com/site/about/guidelines.xhtml

Funding statement

GaPP2 is funded by a grant from the NIHR/MRC Efficacy and Mechanism Evaluation (EME) Programme, Reference: 13/52/04.

Competing interests

KV has received research funding from Pfizer pharmaceuticals and Bayer Healthcare, honoraria from Bayer Healthcare, and consultancy fees from Grunenthal Gmb. AH receives grant funding from the NIHR and the Medical Research Council. APB has no competing issues as no direct funding from industry. APB is currently President of the British pain Society. JB declares no competing interests. SB receives grant funding from NIHR and Chief Scientist Office Scotland. He has been a speaker at a number of conferences that have received funding from the pharmaceutical industry. His clinical colleagues receive industry support for travel and for Departmental Seminars. AW has received a consulting fee from Astellas. GJM is Chief Investigator of the British Society for Rheumatology (BSR) Biologics Register in Ankylosing Spondylitis. This is funded by the BSR who receive funds from Pfizer, AbbVie and UCB. YC receives grant funding from NIHR. RC has secured research funding from Allergan and is a member of Allergan's scientific advisory panel. He also receives grant funding from the NIHR via UCL Biomedical Research Centre. IT is supported by grants from NIHR Oxford Biomedical Research Centre, Medical Research Council of Great Britain and Northern Ireland, the Wellcome Trust.

For peer review only - http://bmjopen.bmj.com/site/about/guidelines.xhtml

Table 1: Dose escalation schedule for GaPP2

Day in study	Total number of capsules/day (maximum)	Dosing	Maximum daily dose of gabapentin
1	1	1 capsule night	300 mg
2	1	1 capsule night	300 mg
3	1	1 capsule night	300 mg
4	2	1 capsule twice daily	600 mg
5	2	1 capsule twice daily	600 mg
6	2	1 capsule twice daily	600 mg
7	3	1 capsule three times daily	900 mg
8	3	1 capsule three times daily	900 mg
9	3	1 capsule three times daily	900 mg
10	4	1 capsule twice + 2 capsules at night	1200 mg
11	4	1 capsule twice + 2 capsules at night	1200 mg
12	4	1 capsule twice + 2 capsules at night	1200 mg
13	5	2 capsules twice + 1 capsule once	1500 mg
14	5	2 capsules twice + 1 capsule once	1500 mg
15	5	2 capsules twice + 1 capsule once	1500 mg
16	6	2 capsules three times daily	1800 mg
17	6	2 capsules three times daily	1800 mg
18	6	2 capsules three times daily	1800 mg
19	7	2 capsules twice + 3 capsules night	2100 mg
20	7	2 capsules twice + 3 capsules night	2100 mg
21	7	2 capsules twice + 3 capsules night	2100 mg
22	8	3 capsules twice + 2 capsules once	2400 mg
23	8	3 capsules twice + 2 capsules once	2400 mg
24	8	3 capsules twice + 2 capsules once	2400 mg
25	9	3 capsules three times daily	2700 mg
26	9	3 capsules three times daily	2700 mg
27	9	3 capsules three times daily	2700 mg
28 - 112	Remain on maximum tolerate dose until the end of week 16. (not exceeding 2700mg or 9 capsules per day). Daily dose should be divided equally into 3 doses.		

For peer review only

For peer review only - http://bmjopen.bmj.com/site/about/guidelines.xhtml

Table 2: Schedule of outcome assessments for GaPP2

**Phase**	**Run-in**	**Baseline, randomisation & treatment dispensed**	**Titration**	**Treatment**		**End of study & unblinding**	**Taper**
**Duration (weeks)**	-4 to -1	0	1-4	5-12	13-16		17-19
Weekly worst and average NRS	x x x x				x x x x		
SF12		X				X	
BPI		X				X	
PCQ		X				X	
SAQ		X				X	
BFI		X				X	
GHQ-12		X				X	
WPAIQ		X				X	
PainDETECT™		X				X	
PUF		X					
Adverse events			X	X	X	X	X
Permitted / Concomitant medication	X		X	X	X		X
Adherence or discontinuation			X	X	X		X

For peer review only

For peer review only - http://bmjopen.bmj.com/site/about/guidelines.xhtml

Figure legend

Figure 1: Study Flow chart.

For peer review only

For peer review only - http://bmjopen.bmj.com/site/about/guidelines.xhtml

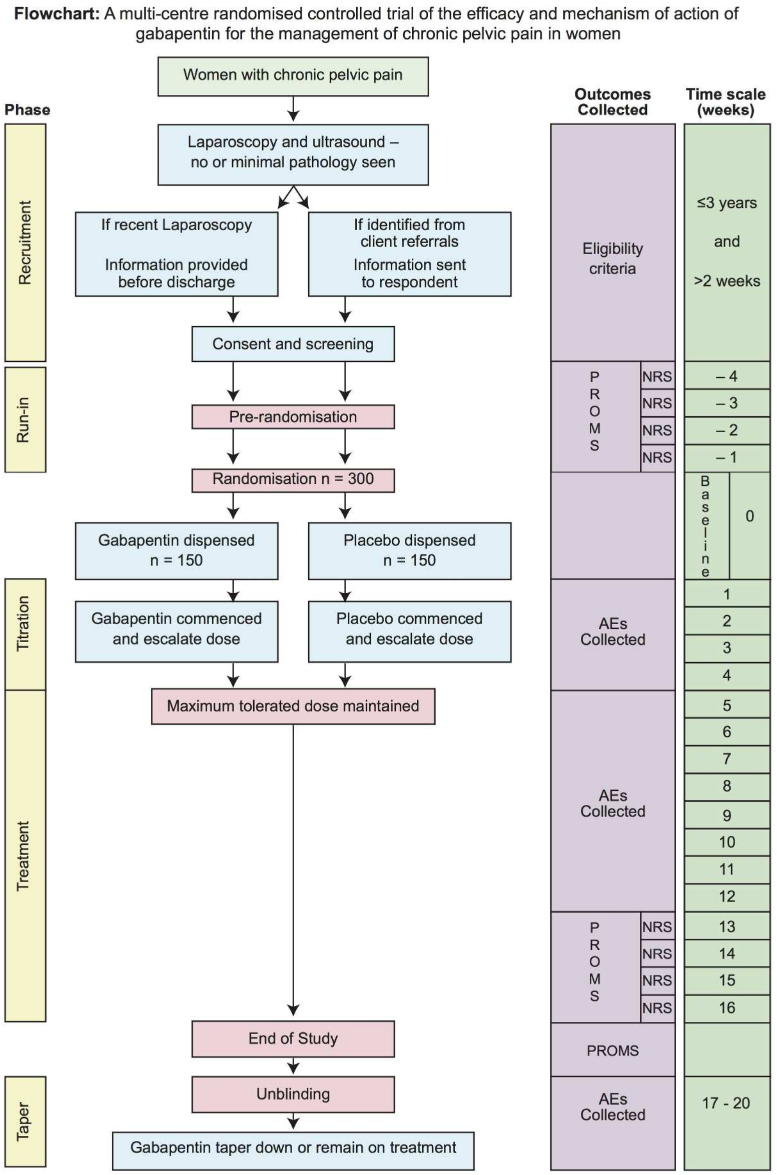

Figure 1

209x296mm (300 x 300 DPI)

STANDARD PROTOCOL ITEMS: RECOMMENDATIONS FOR INTERVENTIONAL TRIALS

SPIRIT 2013 Checklist: Recommended items to address in a clinical trial protocol and related documents*

Section/item	ItemNo	Description	Addressed on page number
**Administrative information**			
Title	1	Descriptive title identifying the study design, population, interventions, and, if applicable, trial acronym	1
Trial registration	2a	Trial identifier and registry name. If not yet registered, name of intended registry	4
	2b	All items from the World Health Organization Trial Registration Data Set	4, 20, 17, 10, 8, 9, 7, 6
Protocol version	3	Date and version identifier	-
Funding	4	Sources and types of financial, material, and other support	20
Roles and responsibilities	5a	Names, affiliations, and roles of protocol contributors	1
	5b	Name and contact information for the trial sponsor	17
	5c	Role of study sponsor and funders, if any, in study design; collection, management, analysis, and interpretation of data; writing of the report; and the decision to submit the report for publication, including whether they will have ultimate authority over any of these activities	17
	5d	Composition, roles, and responsibilities of the coordinating centre, steering committee, endpoint adjudication committee, data management team, and other individuals or groups overseeing the trial, if applicable (see Item 21a for data monitoring committee)	17

For peer review only - http://bmjopen.bmj.com/site/about/guidelines.xhtml

Introduction

Background and rationale	6a	Description of research question and justification for undertaking the trial, including summary of relevant studies (published and unpublished) examining benefits and harms for each intervention	5
	6b	Explanation for choice of comparators	11
Objectives	7	Specific objectives or hypotheses	6
Trial design	8	Description of trial design including type of trial (eg, parallel group, crossover, factorial, single group), allocation ratio, and framework (eg, superiority, equivalence, noninferiority, exploratory)	7-8

Methods: Participants, interventions, and outcomes

Study setting	9	Description of study settings (eg, community clinic, academic hospital) and list of countries where data will be collected. Reference to where list of study sites can be obtained	10
Eligibility criteria	10	Inclusion and exclusion criteria for participants. If applicable, eligibility criteria for study centres and individuals who will perform the interventions (eg, surgeons, psychotherapists)	8-9
Interventions	11a	Interventions for each group with sufficient detail to allow replication, including how and when they will be administered	10
	11b	Criteria for discontinuing or modifying allocated interventions for a given trial participant (eg, drug dose change in response to harms, participant request, or improving/worsening disease)	14
	11c	Strategies to improve adherence to intervention protocols, and any procedures for monitoring adherence (eg, drug tablet return, laboratory tests)	12
	11d	Relevant concomitant care and interventions that are permitted or prohibited during the trial	10

For peer review only - http://bmjopen.bmj.com/site/about/guidelines.xhtml

Outcomes	12	Primary, secondary, and other outcomes, including the specific measurement variable (eg, systolic blood pressure), analysis metric (eg, change from baseline, final value, time to event), method of aggregation (eg, median, proportion), and time point for each outcome. Explanation of the clinical relevance of chosen efficacy and harm outcomes is strongly recommended	6-7
Participant timeline	13	Time schedule of enrolment, interventions (including any run-ins and washouts), assessments, and visits for participants. A schematic diagram is highly recommended (see Figure)	23, 25
Sample size	14	Estimated number of participants needed to achieve study objectives and how it was determined, including clinical and statistical assumptions supporting any sample size calculations	15
Recruitment	15	Strategies for achieving adequate participant enrolment to reach target sample size	9
**Methods: Assignment of interventions (for controlled trials)**			
Allocation:			
Sequence generation	16a	Method of generating the allocation sequence (eg, computer-generated random numbers), and list of any factors for stratification. To reduce predictability of a random sequence, details of any planned restriction (eg, blocking) should be provided in a separate document that is unavailable to those who enrol participants or assign interventions	10
Allocation concealment mechanism	16b	Mechanism of implementing the allocation sequence (eg, central telephone; sequentially numbered, opaque, sealed envelopes), describing any steps to conceal the sequence until interventions are assigned	10
Implementation	16c	Who will generate the allocation sequence, who will enrol participants, and who will assign participants to interventions	10
Blinding (masking)	17a	Who will be blinded after assignment to interventions (eg, trial participants, care providers, outcome assessors, data analysts), and how	10

For peer review only - http://bmjopen.bmj.com/site/about/guidelines.xhtml

	17b	If blinded, circumstances under which unblinding is permissible, and procedure for revealing a participant's allocated intervention during the trial	14
**Methods: Data collection, management, and analysis**			
Data collection methods	18a	Plans for assessment and collection of outcome, baseline, and other trial data, including any related processes to promote data quality (eg, duplicate measurements, training of assessors) and a description of study instruments (eg, questionnaires, laboratory tests) along with their reliability and validity, if known. Reference to where data collection forms can be found, if not in the protocol	11-13, 25
	18b	Plans to promote participant retention and complete follow-up, including list of any outcome data to be collected for participants who discontinue or deviate from intervention protocols	14, 16
Data management	19	Plans for data entry, coding, security, and storage, including any related processes to promote data quality (eg, double data entry; range checks for data values). Reference to where details of data management procedures can be found, if not in the protocol	11
Statistical methods	20a	Statistical methods for analysing primary and secondary outcomes. Reference to where other details of the statistical analysis plan can be found, if not in the protocol	15-17
	20b	Methods for any additional analyses (eg, subgroup and adjusted analyses)	16
	20c	Definition of analysis population relating to protocol non-adherence (eg, as randomised analysis), and any statistical methods to handle missing data (eg, multiple imputation)	16
**Methods: Monitoring**			
Data monitoring	21a	Composition of data monitoring committee (DMC); summary of its role and reporting structure; statement of whether it is independent from the sponsor and competing interests; and reference to where further details about its charter can be found, if not in the protocol. Alternatively, an explanation of why a DMC is not needed	17

	21b	Description of any interim analyses and stopping guidelines, including who will have access to these interim results and make the final decision to terminate the trial	-
Harms	22	Plans for collecting, assessing, reporting, and managing solicited and spontaneously reported adverse events and other unintended effects of trial interventions or trial conduct	14
Auditing	23	Frequency and procedures for auditing trial conduct, if any, and whether the process will be independent from investigators and the sponsor	17
**Ethics and dissemination**			
Research ethics approval	24	Plans for seeking research ethics committee/institutional review board (REC/IRB) approval	17
Protocol amendments	25	Plans for communicating important protocol modifications (eg, changes to eligibility criteria, outcomes, analyses) to relevant parties (eg, investigators, REC/IRBs, trial participants, trial registries, journals, regulators)	17
Consent or assent	26a	Who will obtain informed consent or assent from potential trial participants or authorised surrogates, and how (see Item 32)	9
	26b	Additional consent provisions for collection and use of participant data and biological specimens in ancillary studies, if applicable	-
Confidentiality	27	How personal information about potential and enrolled participants will be collected, shared, and maintained in order to protect confidentiality before, during, and after the trial	11
Declaration of interests	28	Financial and other competing interests for principal investigators for the overall trial and each study site	21
Access to data	29	Statement of who will have access to the final trial dataset, and disclosure of contractual agreements that limit such access for investigators	17
Ancillary and post-trial care	30	Provisions, if any, for ancillary and post-trial care, and for compensation to those who suffer harm from trial participation	14

For peer review only - http://bmjopen.bmj.com/site/about/guidelines.xhtml

Dissemination policy	31a	Plans for investigators and sponsor to communicate trial results to participants, healthcare professionals, the public, and other relevant groups (eg, via publication, reporting in results databases, or other data sharing arrangements), including any publication restrictions	17
	31b	Authorship eligibility guidelines and any intended use of professional writers	17
	31c	Plans, if any, for granting public access to the full protocol, participant-level dataset, and statistical code	-
**Appendices**			
Informed consent materials	32	Model consent form and other related documentation given to participants and authorised surrogates	-
Biological specimens	33	Plans for collection, laboratory evaluation, and storage of biological specimens for genetic or molecular analysis in the current trial and for future use in ancillary studies, if applicable	-

*It is strongly recommended that this checklist be read in conjunction with the SPIRIT 2013 Explanation & Elaboration for important clarification on the items. Amendments to the protocol should be tracked and dated. The SPIRIT checklist is copyrighted by the SPIRIT Group under the Creative Commons "Attribution-NonCommercial-NoDerivs 3.0 Unported" license.

For peer review only

For peer review only - http://bmjopen.bmj.com/site/about/guidelines.xhtml
